# An evolutionary learning and network approach to identifying key metabolites for osteoarthritis

**DOI:** 10.1371/journal.pcbi.1005986

**Published:** 2018-03-01

**Authors:** Ting Hu, Karoliina Oksanen, Weidong Zhang, Ed Randell, Andrew Furey, Guang Sun, Guangju Zhai

**Affiliations:** 1 Department of Computer Science, Memorial University, St. John’s, Newfoundland and Labrador, Canada; 2 Faculty of Medicine, Memorial University, St. John’s, Newfoundland and Labrador, Canada; 3 School of Pharmaceutical Sciences, Jilin University, Changchun, China; CPERI, GREECE

## Abstract

Metabolomics studies use quantitative analyses of metabolites from body fluids or tissues in order to investigate a sequence of cellular processes and biological systems in response to genetic and environmental influences. This promises an immense potential for a better understanding of the pathogenesis of complex diseases. Most conventional metabolomics analysis methods exam one metabolite at a time and may overlook the synergistic effect of combining multiple metabolites. In this article, we proposed a new bioinformatics framework that infers the non-linear synergy among multiple metabolites using a symbolic model and subsequently, identify key metabolites using network analysis. Such a symbolic model is able to represent a complex non-linear relationship among a set of metabolites associated with osteoarthritis (OA) and is automatically learned using an evolutionary algorithm. Applied to the Newfoundland Osteoarthritis Study (NFOAS) dataset, our methodology was able to identify nine key metabolites including some known osteoarthritis-associated metabolites and some novel metabolic markers that have never been reported before. The results demonstrate the effectiveness of our methodology and more importantly, with further investigations, propose new hypotheses that can help better understand the OA disease.

## Author summary

Biomedical research has entered a new era where a large number of molecules and different components in biological systems can be quantitatively examined to investigate the causes of common human diseases. However, given the complexity of biological systems, those causes may not contribute to diseases individually but through interactions. The identification of those interactions, or the synergy of multiple factors, is a very challenging task due to the computational limitation, as well as the lack of effective methodologies for investigating multiple factors simultaneously. In this study, we proposed to model such an interaction effect through a self-learning algorithm using mechanisms inspired by natural evolution. Moreover, by constructing a synergy network using those evolved models, we were able to identify a set of interacting factors associated with a particular disease.

## Introduction

Systems biology is an emerging research direction that takes a holistic approach to modeling complex biological systems [1–3]. It requires multidisciplinary efforts from research fields including biomedicine, statistics, and computer science. Systems biology approaches embrace the complexity of biological systems and focus on modeling the interactions among multiple components in biological systems including genome, transcriptome, proteome, and metabolome [4–8]. By integrating a variety of omics data, systems biology for human disease studies aims at better understanding the etiology of common diseases, discovering biomarkers that can help predict early disease onset, progression and severity, and identifying new drug targets [9, 10].

Integrative data analysis and mining for systems biology often include hundreds to thousands of variables such as genes, proteins, and metabolites [11]. Most conventional tools adopt a univariate analysis strategy and may overlook the intertwined relationships among multiple variables. However, the high dimensionality has imposed a computational challenge for multivariate analyses since searching combinations of variables becomes prohibitive as the search space grows exponentially with the number of variables.

It has brought about the realization that the development and application of powerful informatics and data mining methodologies for systems biology are critical and hold great potentials for the next generation of biomedical research [12, 13]. Machine learning and heuristic search algorithms, including principal component analysis [14], artificial neural networks [15], and random forest [16], have seen increasing and successful applications in omics data mining for biomarker discovery. However, such interdisciplinary research direction is still in a preliminary stage, and more learning and modeling algorithms are yet to be explored and developed in future investigations.

In this article, we developed a new bioinformatics framework for high-dimensionality omics data analysis where we used an evolutionary learning algorithm to discover key metabolites and their combinations for an osteoarthritis (OA) metabolomics study. The non-linear synergistic effects of combining multiple metabolites were inferred using symbolic models that were trained through improving classification accuracies to predict the disease status. The key individual and combinations of synergistic metabolites are further visualized and analyzed using networks.

Evolutionary algorithms define a collection of meta-heuristic optimization and modeling algorithms inspired by natural evolution [17–20]. An evolutionary algorithm maintains a population of diverse candidate solutions, which are compared with the desired outcome. Then, through multiple generations of variation, selection, and reproduction, the population adapts to the selection criterion (the relative distance from the desired outcome), and produces fitter solutions. Evolutionary algorithms are highly robust and powerful in tackling imprecise and incomplete problems, thanks to their automated search mechanisms. They are also extremely parallelizable, due to their distinguishing feature of population-based search, which also allows them to scale to solve large and complex problems. Evolutionary algorithms have been successfully applied to modeling problems, where they can automatically derive a symbolic model of an aggregation of interrelated attributes through an evolutionary training process.

OA is the most common form of arthritis. It causes substantial morbidity and disability in the elderly populations and imposes a great economic burden on our society [21, 22]. Despite a high prevalence and societal impact, there is no medication that can cure it, or reverse or halt the disease progression, partly because its pathogenesis is still unclear and there is no reliable method that can be used for early OA diagnosis. Recent developments in the field of metabolomics provide an array of new tools for the study of OA. Metabolites are intermediate and end products of various cellular processes and their levels of concentration serve as a good indicator of a sequence of biological systems in response to genetic and environmental influences. A large number of small-molecule metabolites from body fluids or tissues can be quantitatively detected simultaneously, which promises an immense potential for early diagnosis, therapy monitoring and understanding the pathogenesis of complex diseases [23–25].

Our evolutionary algorithm and network analysis were able to identify nine key metabolites that appear most frequently in the best evolved models for predicting the disease outcome, four of which also serve as hubs and bottlenecks in the metabolite synergy network. Some of the nine metabolites were previously found highly associated with OA, and the rest are novel findings that could be very useful proposing new hypothesis to better understand the disease.

## Methods

### Ethics statement

The study protocol was approved by the Health Research Ethics Authority (HREA) of the province of Newfoundland and Labrador, Canada, with reference number 11.311 and a written consent was obtained from all the participants.

### Osteoarthritis metabolomics data

In the OA dataset used for the current study, knee OA patients were selected from the Newfoundland Osteoarthritis Study (NFOAS) initiated in 2011 [26]. The NFOAS aimed at identifying novel genetic, epigenetic, and biochemical markers for OA. The NFOAS recruited OA patients who underwent a total knee replacement surgery due to primary OA between November 2011 and December 2013 at the St. Clare’s Mercy Hospital and Health Science Centre General Hospital in St. John’s, the capital city of Newfoundland and Labrador (NL), Canada. Healthy controls were selected from the CODING study (The Complex Diseases in the Newfoundland population: Environment and Genetics), where participants were adult volunteers [27].

Both cases and controls were from the same source population of Newfoundland and Labrador. Knee OA diagnosis was made based on the American College of Rheumatology clinical criteria for the classification of idiopathic OA of the knee [28] and the judgment of the attending orthopedic surgeons. Controls were individuals without self-reported family doctor diagnosed knee OA based on their medical information collected by a self-administered questionnaire. We collected 153 OA cases and 236 healthy controls.

Blood samples were collected after at least 8 hours of fasting and plasma was separated from blood using the standard protocol. Metabolic profiling was performed on plasma using the Waters XEVO TQ MS system (Waters Limited, Mississauga, Ontario, Canada) coupled with Biocrates AbsoluteIDQ p180 kit, which measures 186 metabolites including 90 glycerophospholipids, 40 acylcarnitines (1 free carnitine), 21 amino acids, 19 biogenic amines, 15 sphingolipids and 1 hexose (above 90 percent is glucose). The details of the 186 metabolites and the metabolic profiling method were described in our previous publication [29]. Over 90% of the metabolites (167/186) were successfully determined in each sample.

Prior to performing the informatics analyses, several steps of preprocessing were applied to the dataset. Batch correction was performed by multiplying each metabolite concentration value by the ratio of the overall mean and the batch mean for that metabolite. Then, covariate adjustment was performed to remove the variation due to individual’s age, gender, and body mass index (BMI). The samples were randomly assigned to either a discovery or replication dataset, such that cases and controls were divided evenly between the two datasets. Finally, each metabolite concentration value was normalized to zero mean and unit variance across the population.

### Evolutionary learning algorithm

In this study, the algorithm used to model the non-linear synergy among multiple metabolites associated with OA is a branch of evolutionary computation, termed genetic programming [30]. A population of diverse candidate prediction models is generated randomly in the step of initialization and will evolve to improve prediction accuracy gradually through a number of generations. After evolution halts, the best model of the population in the final generation will be the output.

Each candidate prediction model takes the form of a symbolic computer program comprised of a set of sequential instructions. An instruction can be an assignment statement or a conditional statement. The conditional if instructions affect the program flow such that the instruction immediately following the if instruction is not executed if the condition is false. In the case of nested if instructions, each of the successive conditions needs to be true in order for the instruction following the chain of if instructions to be executed.

A register r stores the value of a feature, a calculation variable, or a constant. A feature can be a predictor or an attribute used to make a prediction of the outcome. In the context of the current study, features are concentration levels of metabolites in the samples. A calculation variable serves as a temporary buffer that enhances the computation capacity. In an assignment instruction, only registers storing calculation variables can serve as the return on the left side of the assignment symbol “=”, but any register can serve as an operand on the right-hand side. This is to prevent overwriting the feature values. When a prediction model is evaluated on a given sample, feature registers take all the values of the sample, and the set of instructions are executed sequentially. The sigmoid transformation of the final value stored in the designated calculation register r[0] is used to predict the outcome of the sample, i.e., if *S*(r[0]) is greater than or equal to 0.5, the sample is predicted as diseased (class one), otherwise the sample is predicted as healthy (class zero).

An example of classification model with eight instructions is given below. Here, the output register r[0] and calculation registers r[4] and r[5] are all initialized with ones. Feature registers r[1-3] take input values from three metabolite concentration levels m[1-3] respectively. For instance, when a sample with m[1-3] values as {0.2, 0.01, 0.085} is input to this classification model, the conditional statement r[1]>r[3] in instruction I1 becomes true, so in instruction I2, r[0] changes its value to 0.51. The rest of the instructions are executed sequentially, and the final value of r[0] is set to 1.0039. Its sigmoid transformation *S*(1.0039) is greater than 0.5, so this sample will be classified by this model as class one, i.e., diseased.

I1: if r[1]> r[3]

I2:   then r[0] = r[2] + 0.5

I3: r[4] = r[2] / r[0]

I4: if r[0] > 4

I5:   then if r[3] < 10

I6:        then r[5] = r[3]—r[4]

I7: r[4] = r[4] * r[1]

I8: r[0] = r[5] + r[4]

At the initial generation, a population of diverse classification models was generated randomly. The fitness of each model was evaluated using mean classification error (MCE), computed as the average number of incorrectly classified training samples. A set of models were chosen as parents based on their fitness, and variation operators, including mutation and recombination, were applied to them. A mutation alters an element of a randomly picked instruction, i.e., replacing a return or an operand register by a randomly generated one or replacing the operator. Recombination swaps segments of instructions of two parent models. Survival selection picks fitter models to form the population for the next generation. Such an evolution process iterates for a certain number of generations, and the model with the lowest MCE at the end is output as the final best model of a run.

This evolutionary modeling algorithm was implemented using the Julia programming language [31]. The main parameters used in the implementation are shown in Table 1. A five-fold cross-validation was used to prevent overfitting so that each run of the algorithm produced five best classification models as its output.

**Table 1:** Parameters used in the evolutionary modeling algorithm.

Fitness function	Mean classification error (MCE)
Program initialization	Random
Program length	[1, 500]
Population size	500
Number of parents	500
Parent selection	Tournament with size 16
Survival selection	Truncation
Number of generations	500
Operator set	{+, −, ×, ÷, *x*^*y*^, if <, if >}
Constant set	{1, 2, 3, 4, 5, 6, 7, 8, 9, 10}
Calculation registers	150
Mutation operators	Effective instructions only

For the first round of analysis, the evolutionary learning algorithm was run on the discovery dataset using 200 distinct seed values for the random number generator. As a result of the cross validation, our implementation gave five different best classification models for each seed value, resulting in a total of 1000 best classification models.

We investigated the resulting classification models by calculating various statistics of the fitness (MCE) values, sensitivity, specificity and area under the curve (AUC) as computed on the testing fold for each run. In addition, we inspected the models by counting how often each of the 167 metabolites appeared as predictive variables in the set of 1000 best models.

### Metabolite synergy network

In addition to looking at the individual occurrence of single metabolites in the best classification models, the co-occurrence of metabolites in the models was studied by counting the number of times each metabolite pair appeared together in the same model. The top 1% of the resulting metabolite pairs, ranked by decreasing frequency, were used to construct a metabolite synergy network. Network science has seen increasing applications in biomedical research [32–34], where biological entities are represented as vertices and their relationships can be modeled using edges linking pairs of vertices. Network modeling is a powerful tool to study interconnections among a large number of biological entities. In this study, vertices represent metabolites and an edge links two metabolites if they have a co-occurrence frequency in the set of 1000 best prediction models greater than the given cutoff. The network was rendered and analyzed using the Cytoscape software [35].

For the second round of a more focused analysis, only the subset of metabolites appearing in the metabolite synergy network was used as a restricted feature set in a repeated model learning implementation, allowing the evolutionary algorithm to only use these more important metabolites to construct the classification models. The analysis was performed on both the discovery and replication datasets, each resulting in another set of 1000 best classification models. The intersection of the top 20 most common metabolites from the discovery and replication runs was reported, and such metabolites are regarded interacting metabolites with high potential associations to the disease of OA.

To evaluate the classification power of the best models found using our evolutionary algorithm, we trained logistic regression on both the discovery and replication datasets using the reduced feature set and compared its classification performance with our evolutionary algorithm.

## Results

### Classification models evolved on the full feature set

In the first round of analysis on the discovery dataset, the full feature set of 167 metabolites was used for the evolutionary algorithm to search for the best classification models. The classification performance of those best models was then evaluated using testing samples. Statistics for the results are shown in Table 2. It can be seen that the best results, among them the lowest MCE and the highest AUC value, suggest that some of the classification models found with the evolutionary algorithm during this first full feature scenario already achieve a reasonably high prediction accuracy.

**Table 2:** Statistics of the results on the full feature set (discovery).

	MCE	Sensitivity	Specificity	AUC
Mean	0.367	0.684	0.584	0.663
Median	0.367	0.667	0.600	0.667
Min	0.067	0.200	0.200	0.320
Max	0.667	1.000	0.933	0.947
Std dev	0.095	0.146	0.142	0.110
5% confidence	0.181	0.398	0.305	0.447
95% confidence	0.553	0.970	0.862	0.879

Although a feature register can be any of the full set of 167 metabolites in the NFOAS dataset, the final best models usually only contain a subset of those features, given the nature of the evolutionary algorithm. Moreover, recall that since the final value stored in the designated output register is used to compute the classification outcome, some instructions can be redundant and not have any effect on the output value. Those input features that appear in effective instructions, that is, instructions which do contribute to the outcome, are considered effective features.

**Fig 1:**
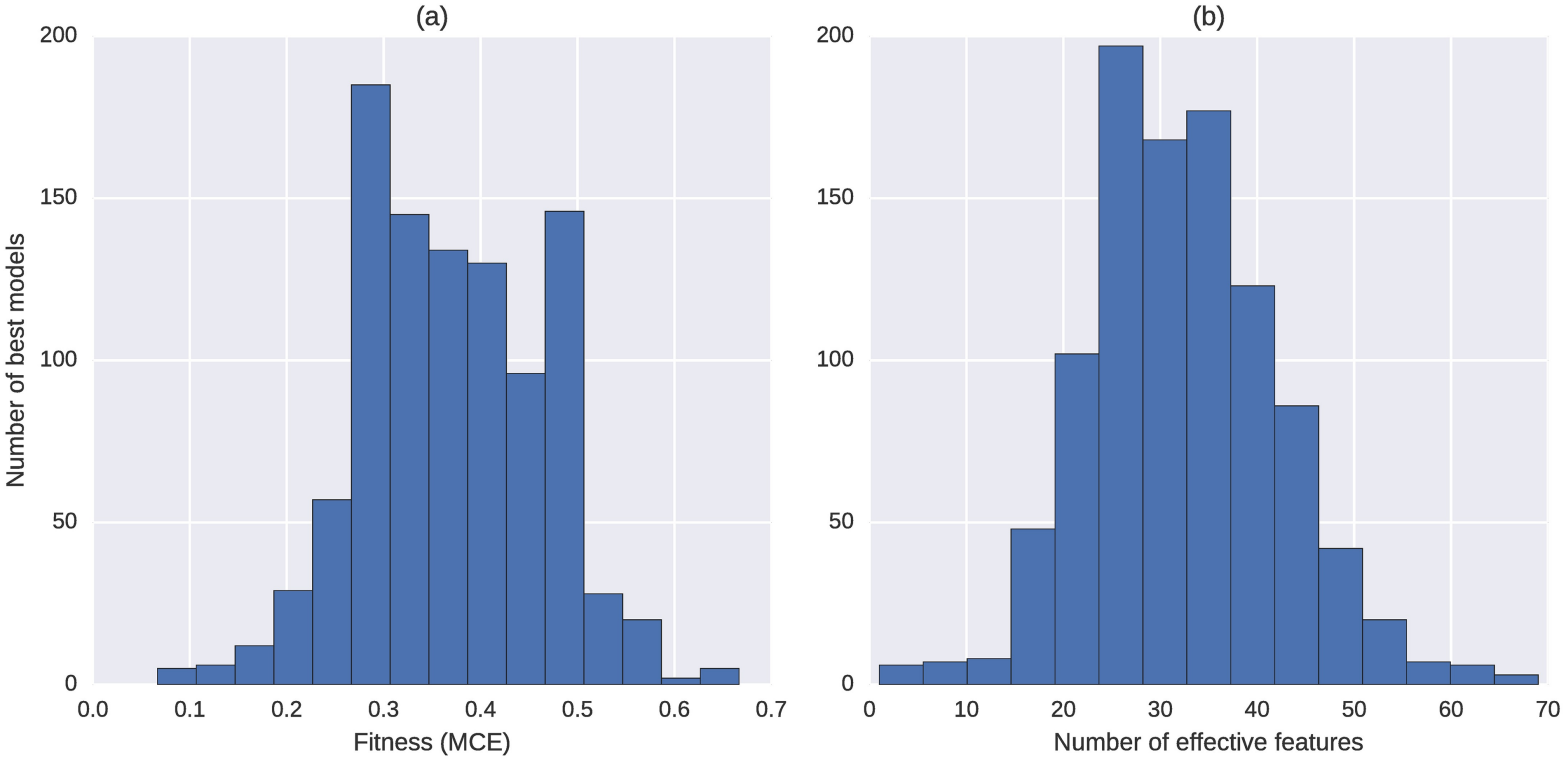
Distributions of the (a) fitness (MCE) and (b) number of effective features for the 1000 best models, full feature set (discovery).

We looked into the number of effective features in the 1000 best models. A visualization of the distributions for fitness (MCE) and the number of effective features can be seen in Fig 1. Most best models have their MCE values between 0.3 and 0.5, while some runs can yield models with a classification error less than 0.1. Most best models include around 20 to 40 effective features, about 10% to 20% of the total feature set. In addition, there turns out to be no correlation between the fitness and number of effective features of the classification models, with Pearson’s correlation coefficient being 0.044 and the associated *p*-value 0.16 (S1 Fig).

**Fig 2:**
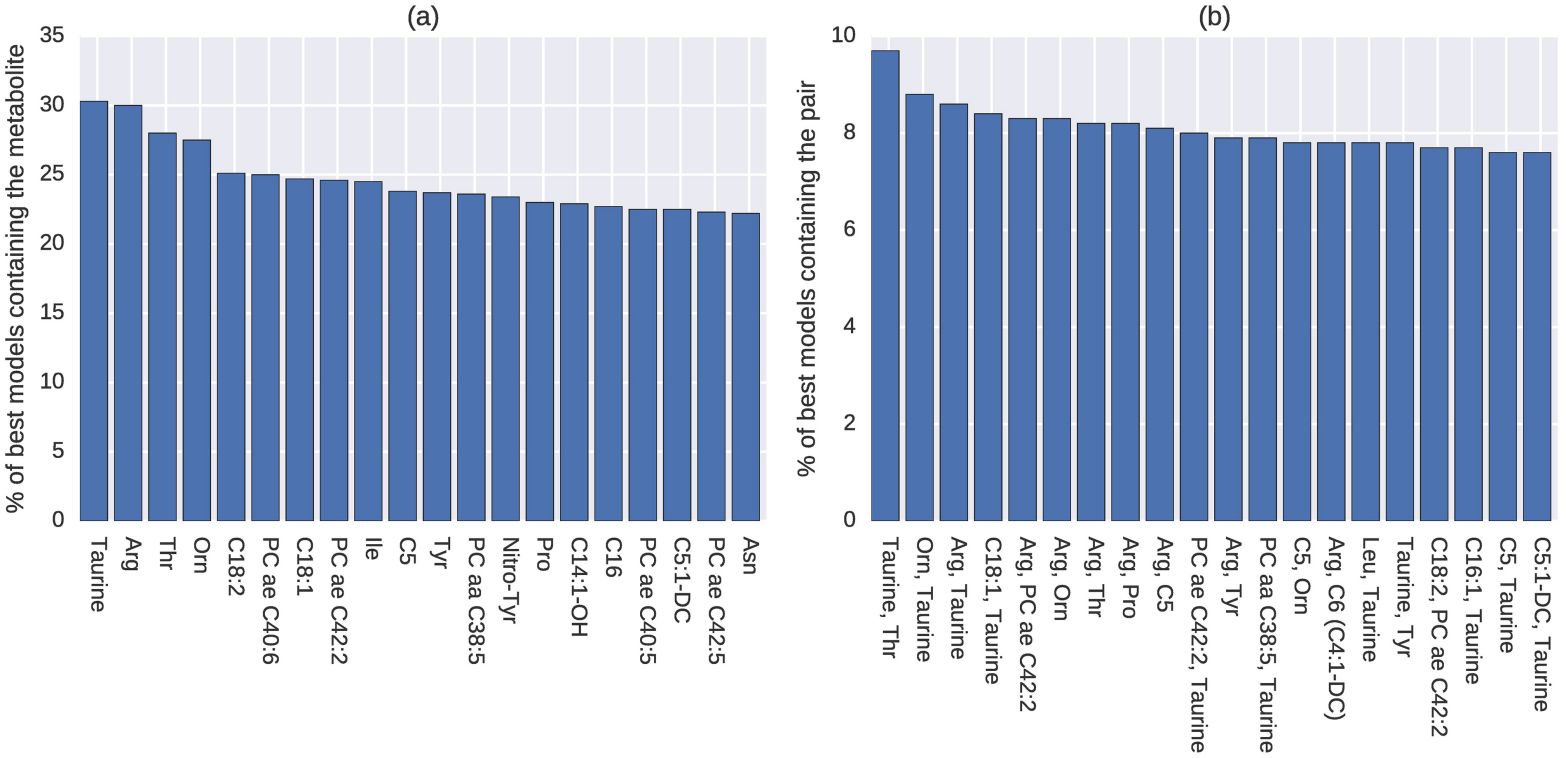
Most common (a) individual metabolites and (b) pairs appearing in the 1000 best models, full feature set (discovery).

The top 20 metabolites and metabolite pairs most commonly contained in the 1000 best models are shown in Fig 2. The top two individual metabolites *taurine* and *arginine* appear in about 30% of the models, with *threonine* and *ornithine* also being found in over 25% of the models. In addition to the highest individual appearance, *taurine* pairs up with *threonine* in about 10% of the best models, and with *ornithine* or *arginine* in about 9% of the best models.

### Network of the top co-occurring metabolites

**Fig 3:**
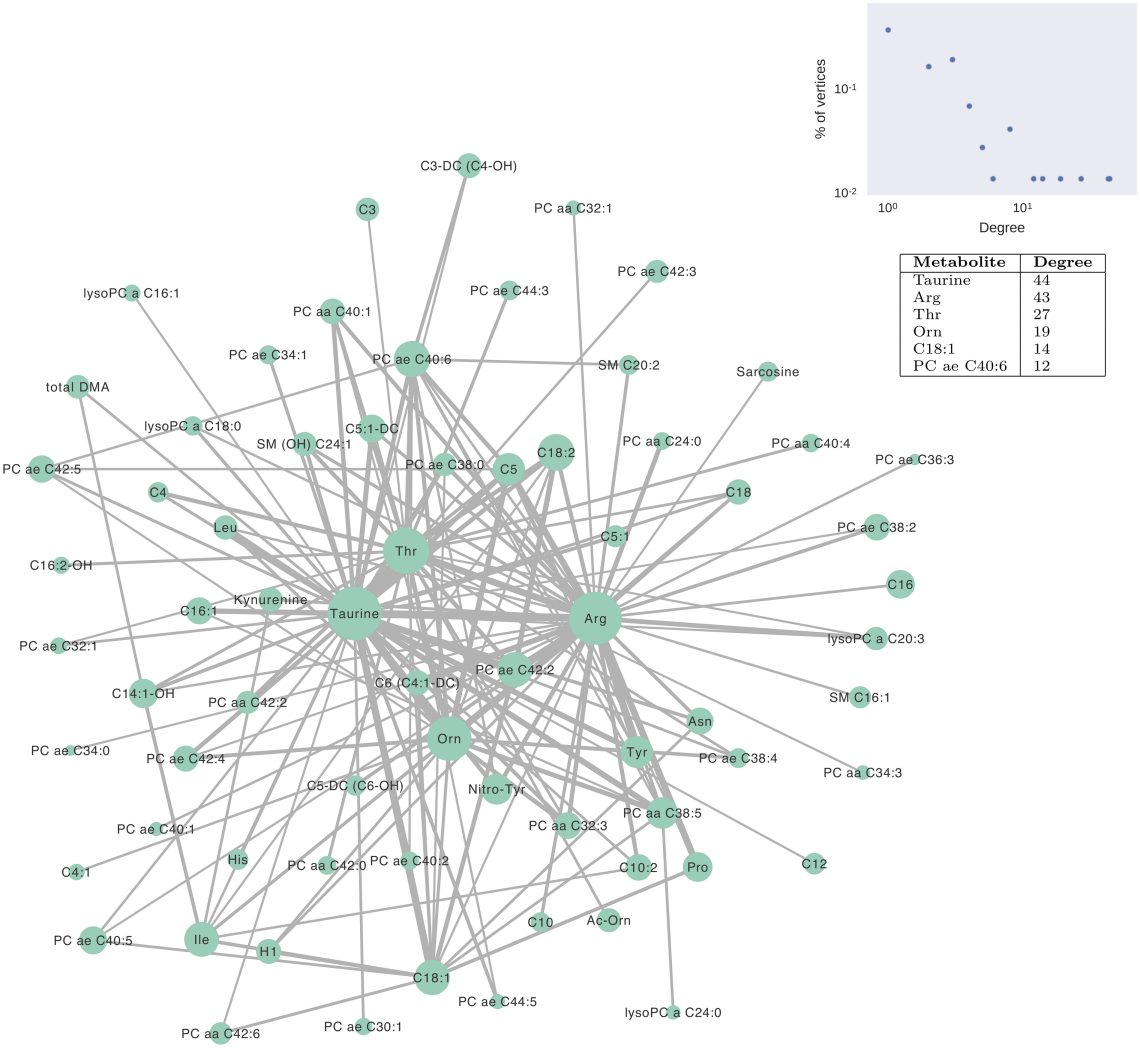
Network of the top 1% most common metabolite pairs, full feature set (discovery). Each vertex is a metabolite and its size is proportional to its corresponding metabolite’s individual occurrence frequency in the best models. An edge links two metabolites if their co-occurrence frequency in the best models is among the top 1% of all the pairs. The edge width is proportional to the co-occurrence frequency. The inset figure shows the degree distribution of this network, and the inset table lists the metabolites that have degrees higher than ten.

The top 1% metabolite pairs out of all $\left(\begin{array}{c} 167\\ 2 \end{array}\right)$ possible combinations were used to construct the network of Fig 3. Here each vertex is a metabolite and an edge links two metabolites if they have a high pairwise occurrence (top 1%) in the best models. The vertex size denotes the frequency of the individual metabolite’s occurrence in the best models, while the edge width shows the frequency of the occurrence of the corresponding metabolite pair. The network has 70 metabolites and 156 edges. There is one connected component and each vertex has an average of 4.5 connected neighbors.

The vertex degree of the network follows a heavy-tail distribution (Fig 3 inset). The metabolites that are individually most common in the best models also make up the vertices with the highest degree in the network, due to the methodology being used. *Taurine* has the highest degree of 44, followed by *arginine* with a degree of 43. *Threonine* and *ornithine* have degrees of 27 and 19 respectively. These four metabolites are also connected to each other in the network, forming a dense core of the network. In contrast, most peripheral vertices have degrees less than four. Those four vertices not only have the highest degrees but also the highest closeness and betweenness centralities, i.e., they serve as essential hubs and bottlenecks of the network.

### Classification models evolved on the reduced feature set

The 70 metabolites appearing in the network of Fig 3, which make up 1% of the most common metabolite pairs in the models for the first round of analysis, were used as the feature set for repeated evolutionary algorithm runs on both of the discovery and replication datasets. The statistics for the discovery dataset run are shown in Table 3, and for the replication dataset in Table 4. Comparing these statistics to those for the full feature set runs in Table 2, it can be seen that the AUC value for the reduced feature set is higher on both the discovery and replication datasets. Thus the corresponding models achieve better performance in predicting the presence of OA based on the metabolite data.

**Table 3:** Statistics of the results on the reduced feature set (discovery).

	MCE	Sensitivity	Specificity	AUC
Mean	0.325	0.723	0.628	0.704
Median	0.333	0.733	0.600	0.709
Min	0.100	0.267	0.200	0.362
Max	0.600	1.000	1.000	1.000
Std dev	0.089	0.135	0.141	0.103
5% confidence	0.151	0.459	0.353	0.503
95% confidence	0.498	0.987	0.904	0.906

**Table 4:** Statistics of the results on the reduced feature set (replication).

	MCE	Sensitivity	Specificity	AUC
Mean	0.295	0.733	0.678	0.725
Median	0.300	0.733	0.667	0.739
Min	0.033	0.267	0.200	0.380
Max	0.600	1.000	1.000	1.000
Std dev	0.098	0.139	0.162	0.120
5% confidence	0.102	0.460	0.361	0.491
95% confidence	0.488	1.005	0.995	0.959

**Fig 4:**
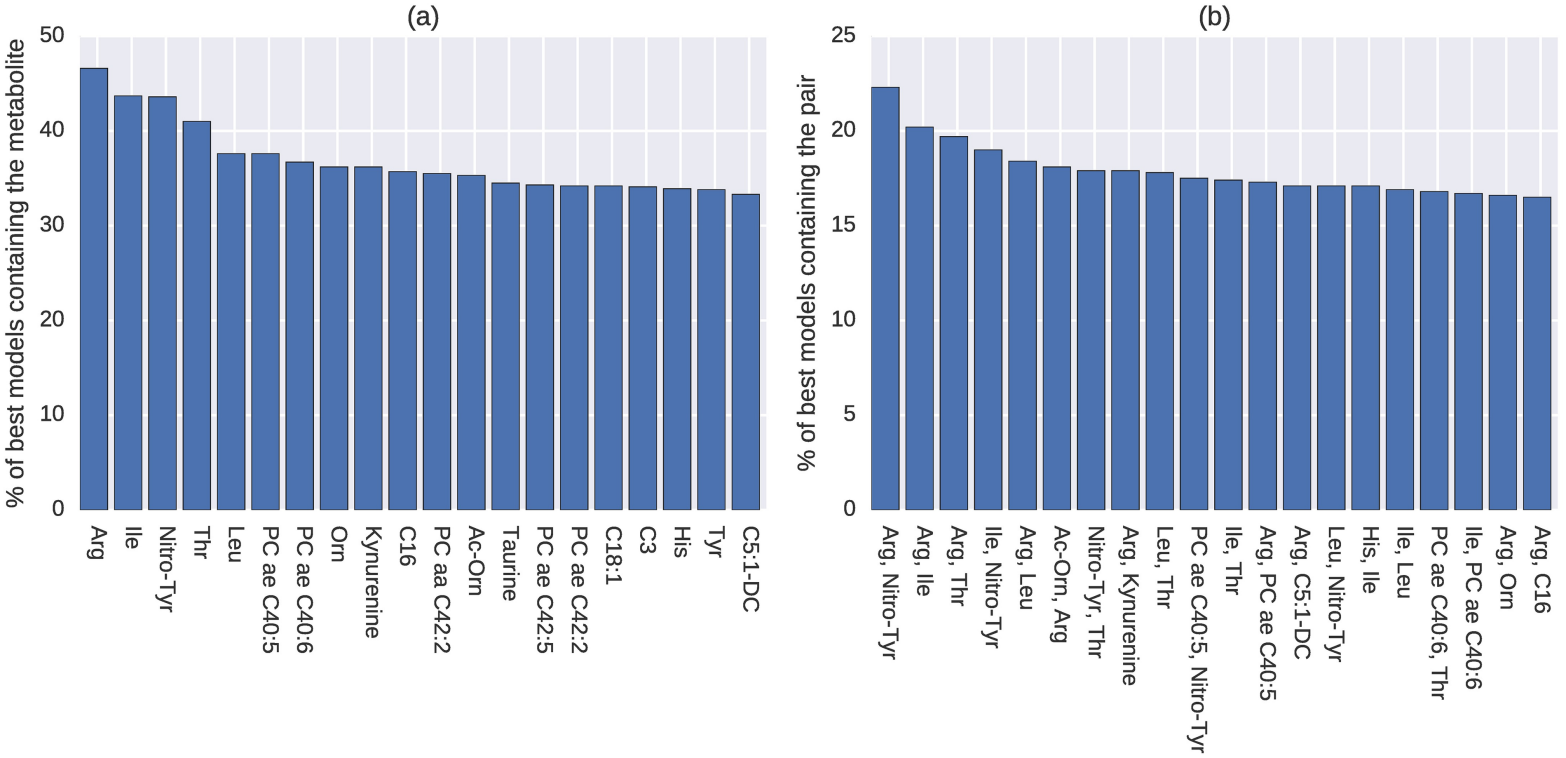
Most common (a) individual metabolites and (b) pairs appearing in the best models, reduced feature set (discovery).

**Fig 5:**
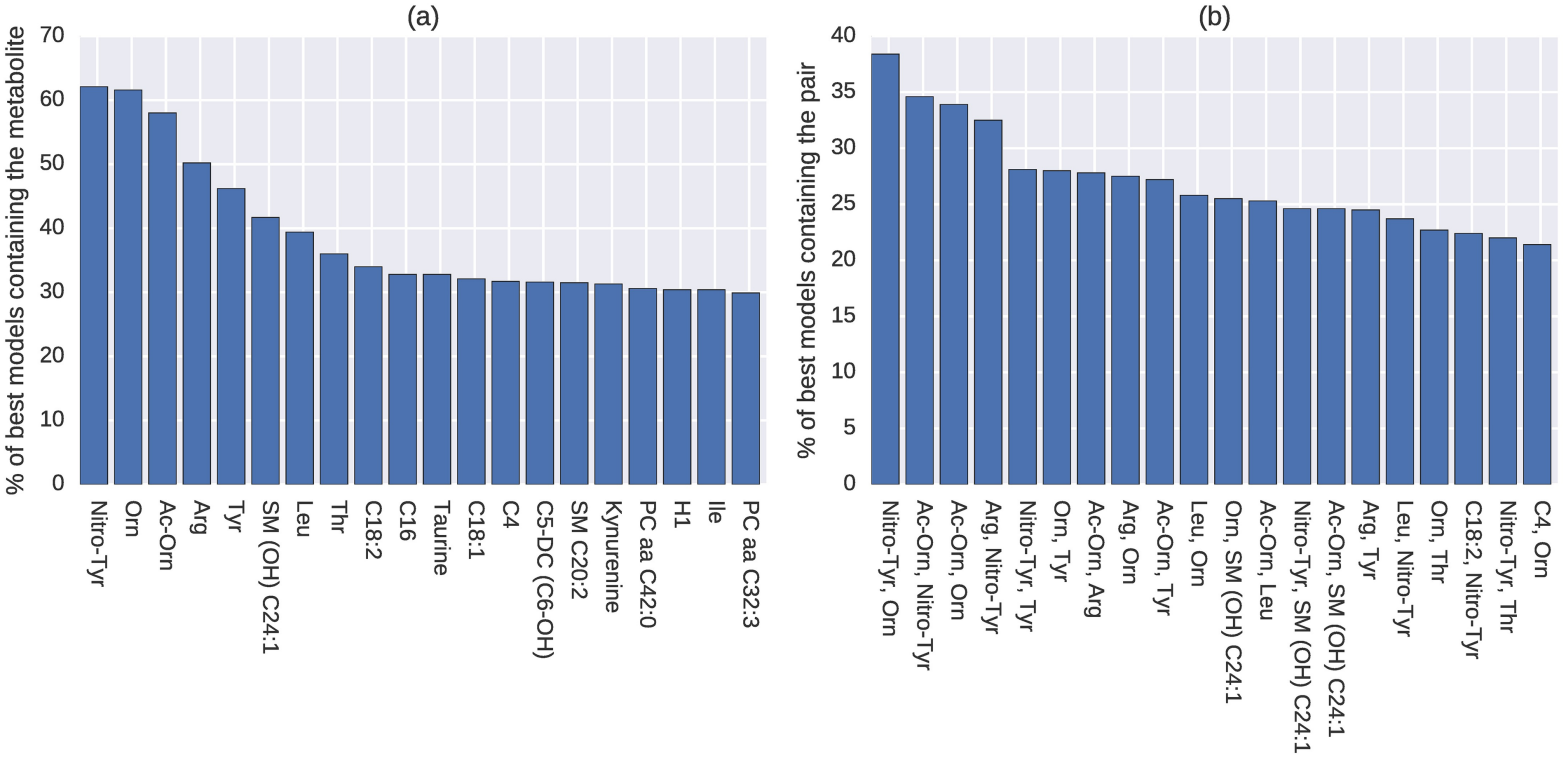
Most common (a) individual metabolites and (b) pairs appearing in the best models, reduced feature set (replication).

The 20 most common metabolites and metabolite pairs for the discovery and replication datasets, using the reduced feature set, are shown in Figs 4 and 5 respectively. The overlap between the 20 most common metabolites from the analysis using the full feature set is notable, with 14/20 and 10/20 metabolites being the same when comparing the full feature set runs to the reduced feature set runs on the discovery and replication datasets respectively. There are 9 metabolites that appear within the 20 most common ones on all three rounds of analyses: *arginine*, *C16*, *C18:1*, *isoleucine*, *nitrotyrosine*, *ornithine*, *taurine*, *threonine*, and *tyrosine*. This set also includes the four top hub and bottleneck metabolites in the previous network (Fig 3).

**Fig 6:**
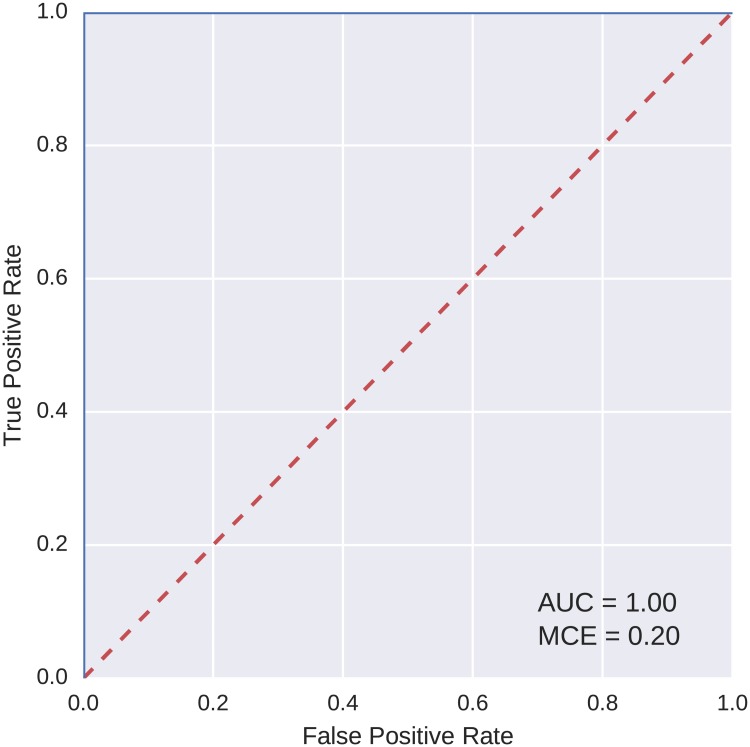
ROC curve for the best model found on the reduced feature set (discovery).

**Fig 7:**
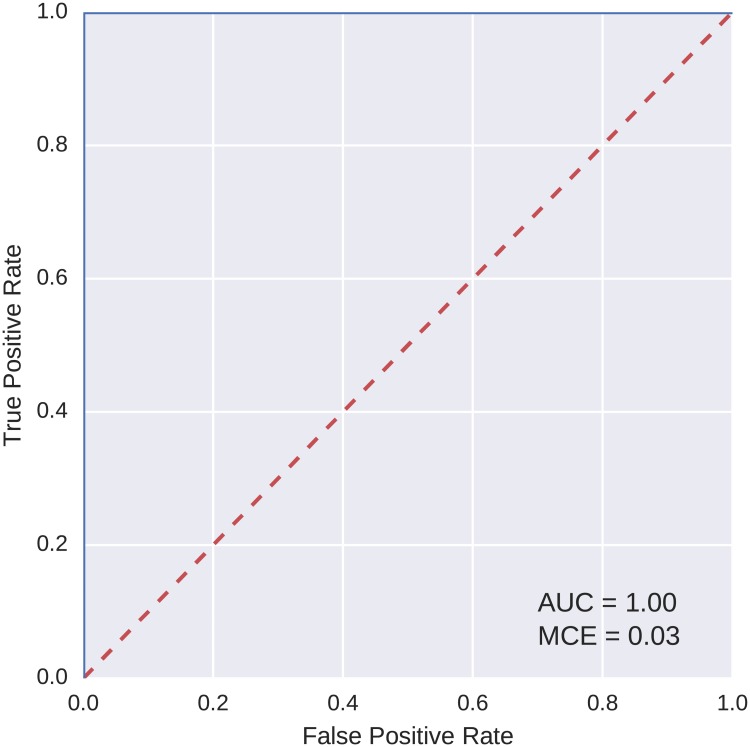
ROC curve for the best model found on the reduced feature set (replication).

The best model based on the AUC value was selected from the models found on each of the discovery and replication datasets. The ROC curves, as computed on the testing fold, for two sample best models are shown in Figs 6 and 7. Both of these models achieved a perfect AUC value of 1.

Pseudocode representations of these two best models are shown in Listings 1 and 2. Here each line contains one instruction, and the instructions are executed one after another like in any imperative language. The r[N] notation denotes calculation register at index *N*, and r[0] is treated as the output register. Calculation registers do not take input from feature values of training or testing data samples, but serve as buffers in the program to enhance its computational capacity. All calculation registers and the output register are initialized with ones at the start of the program’s implementation.

After the program has been run on a data sample, the value contained in the register r[0] is converted to either zero or one, representing the prediction of healthy and diseased individuals respectively, by using the Sigmoid function and rounding to the nearest integer. This value is the classification prediction of the program.

**Listing 1** Pseudocode representation for the best model found on the reduced feature set (discovery).

if r[106] > Orn

  if PC aa C24:0 < r[65]

    r[68] = PC ae C40:5 + PC ae C44:5

r[108] = r[130] / Asn

if PC ae C44:5 > SM (OH) C24:1

  r[108] = lysoPC a C24:0 - r[18]

if r[108] < r[133]

  r[98] = r[87] - Arg

r[51] = PC ae C44:3 + Arg

if Leu > Kynurenine

  r[68] = C5:1 ^ Taurine

r[131] = Nitro-Tyr + r[6]

r[125] = r[68] + r[51]

if C5-DC (C6-OH) > r[33]

  r[98] = r[130] + C4

if PC ae C38:0 > r[131]

  r[98] = PC aa C32:1 * PC ae C40:1

if PC aa C34:3 < 4.0

  r[0] = r[98] - r[125]

**Listing 2** Pseudocode representation for the best model found on the reduced feature set (replication).

r[15] = SM C16:1 ^ r[19]

if PC aa C40:4 < r[15]

  if r[36] > C4

    r[96] = r[125] * SM (OH) C24:1

r[59] = r[80] + Orn

if r[61] < C4

  r[59] = r[72] / r[127]

if C5-DC (C6-OH) > Orn

  r[31] = r[59] - r[117]

r[0] = r[31] - Arg

r[59] = 10.687 / Nitro-Tyr

if Ac-Orn > r[96]

  r[0] = r[59] - 10.595

### Comparison with logistic regression

**Fig 8:**
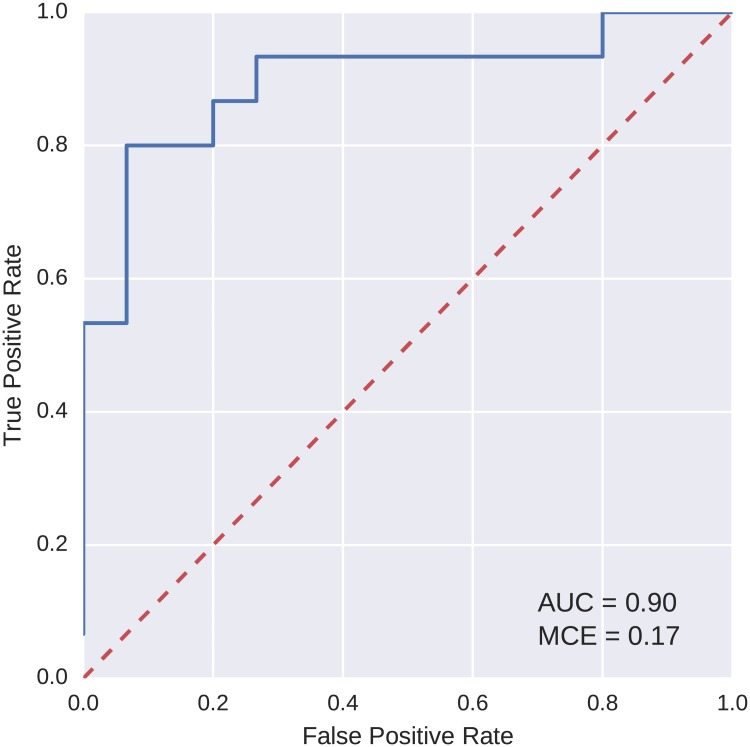
ROC curve for logistic regression, the best model found on the reduced feature set (discovery).

**Fig 9:**
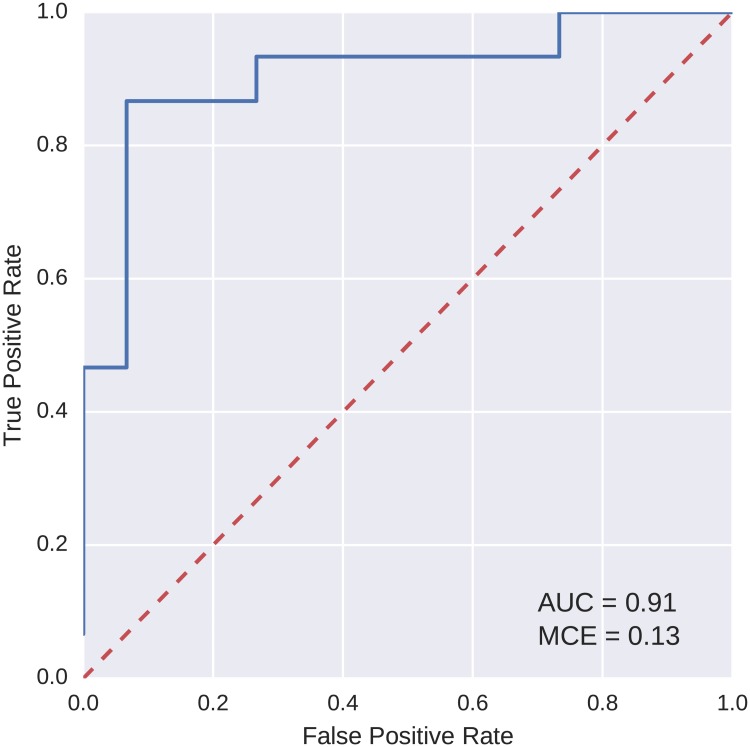
ROC curve for logistic regression, the best model found on the reduced feature set (replication).

To compare the classification power of the best models found using our evolutionary algorithm with a more widely used method, logistic regression was trained on the data. We used the logistic regression implementation from the scikit-learn Python library [36]. The same five-fold cross validation scheme and partitioning of data into the folds were used as with the evolutionary algorithm. ROC curves for the two best classification models found with logistic regression, one for each of the discovery and replication datasets, are shown in Figs 8 and 9. As is apparent from the curves, the AUC values for these models are lower than of those evolved using the evolutionary algorithm.

## Discussion

The fast developing biomedical and computing technologies have brought research to a new era where multi-omics data are produced and mined in order to search for biomarkers of common human diseases. These omics data usually include hundreds to thousands of attributes and such high dimensionality has imposed a great computational challenge for bioinformatics studies. Most existing analyses look at one attribute at a time since the exponential increase of the possible combinations of attributes renders the exhaustive search prohibitive. Such a strategy, however, may overlook important interactions among multiple attributes with limited individual marginal effects.

In this study, we developed an informatics framework that uses an evolutionary algorithm and network analysis to identify both the individual and combinations of metabolites associated with the risk of osteoarthritis (OA). The evolutionary algorithm automatically searches for the models that can best predict the clinical outcome of OA using a feature set of metabolite concentration levels in healthy and diseased samples. Such an automatic learning algorithm performs feature selection systematically through the search for the best classification models and requires minimal prior assumptions on the models. The stochastic and population-based nature of the evolutionary algorithm produces a set of best models. Based on those best models, we constructed a metabolite network where vertices are high association metabolites and vertex sizes reflect their frequencies of occurrences in the best classification models. Edges link pairs of metabolites and their widths represent the co-occurrence frequencies of metabolite pairs. Such a network captures both the most important individual and combinations of metabolites associated with the disease. Moreover, it depicts the interconnected structure and patterns of multiple metabolites, and helps identify metabolic functions that may play a key role in explaining the OA disease.

In the first round of analysis, the entire set of 167 metabolites in the metabolomics study was used for the evolutionary algorithm to search for the models that can best predict the disease outcome. The algorithm was run 200 times and a five-fold cross validation was used to avoid overfitting. Each run produced five best classification models on the five testing datasets, and thus a total number of 1000 best models were generated. The most frequent individual and pairs of metabolites that appear in those best models were reported (Fig 2). In the constructed metabolite network (Fig 3), four key metabolites *taurine*, *arginine*, *threonine*, and *ornithine* were identified as hubs and bottlenecks of the network, which indicates their important role in explaining the disease of OA.

In the second round of analysis, we performed a more focused model search by using only the 70 metabolites included in the network as the reduced feature set. The evolutionary algorithm was executed again on the reduced feature set on both of the discovery and replication datasets. This round of analysis yielded improved classification models with higher prediction accuracies (Tables 3 and 4). Nine metabolites, *arginine*, *C16*, *C18:1*, *isoleucine*, *nitrotyrosine*, *ornithine*, *taurine*, *threonine* and *tyrosine*, were found most frequently appearing in the best models in the discovery dataset as the result of both rounds of analyses and were successfully replicated using the replication dataset, including the previous four key metabolites identified in the network.

The results are interesting as *arginine* and its pathway related metabolites, such as *ornithine*, have been identified as being associated with OA in our previous analysis using traditional methods including pairwise comparison and regression technique [37]. Similarly, *isoleucine* was also previously identified as OA-associated metabolite [38]. The current analyses applied a novel analytic method, the evolutionary algorithm, which confirmed our previous findings and also identified additional novel metabolic markers for OA. These included four amino acids and two acylcarnitines, which could have potential utility in the clinical management of OA. For example, *taurine* is the most abundant free amino acid in humans, and may play an important role in inflammation associated with oxidative stress [39]. It has been reported to be associated with rheumatoid arthritis [40]. *Nitrotyrosine* is also associated with oxidative damage and has been found associated with aging and the development of OA in cartilage samples from both monkeys and humans [41]. The findings in the current study certainly warrant further investigation of the role of those novel metabolic markers in OA.

Our bioinformatics framework, which uses an evolutionary algorithm and network analysis to identify key biomarkers for metabolomics studies, demonstrates the great potential of applying advanced computational techniques to biomedical data mining and model searching problems. Comparing to logistic regression, one of the most commonly used algorithms for such problems, our method was shown to be able to achieve better classification accuracies. Apart from most existing algorithms where metabolites are evaluated individually, our algorithm is able to examine which combinations of multiple metabolites can best predict the disease outcome. Some of the nine key metabolites reported in this study have very limited individual marginal effects, and could be overlooked using the traditional univariate analyses (S2–S10 Figs). In addition, feature selection is embedded in our algorithm, so the search for the most relevant metabolites is systematically performed while evolving the best classification models. Classification models are represented as symbolic relationships between the metabolite concentration levels and the prediction outcome. Such a representation requires minimal prior assumptions on the models and can describe highly complex non-linear relationships. This can be even more important when multiple types of omics data are used in integrated analyses. Our methodology can be a very useful addition to the toolkit for bioinformatics research, and we expect to extend the applications of our methodology to a large range of data and problems in systems biology research.

## Supporting information

S1 Fig The correlation of fitness (MCE) and the number of effective features in the 1000 best models (discovery). (PDF)

S2 Fig The distribution of *arginine* concentration in cases and controls. (PDF)

S3 Fig The distribution of *C16* concentration in cases and controls. (PDF)

S4 Fig The distribution of *C18:1* concentration in cases and controls. (PDF)

S5 Fig The distribution of *isoleucine* concentration in cases and controls. (PDF)

S6 Fig The distribution of *nitrotyrosine* concentration in cases and controls. (PDF)

S7 Fig The distribution of *ornithine* concentration in cases and controls. (PDF)

S8 Fig The distribution of of *taurine* concentration in cases and controls. (PDF)

S9 Fig The distribution of of *threonine* concentration in cases and controls. (PDF)

S10 Fig The distribution of *tyrosine* concentration in cases and controls. (PDF)
