# Supplementary figures and images for: An evolutionary learning and network approach to identifying key metabolites for osteoarthritis
Source: PLoS Comput Biol. 2018 Mar 1;14(3):e1005986. doi: 10.1371/journal.pcbi.1005986 (PMC5849325; doi:10.1371/journal.pcbi.1005986)

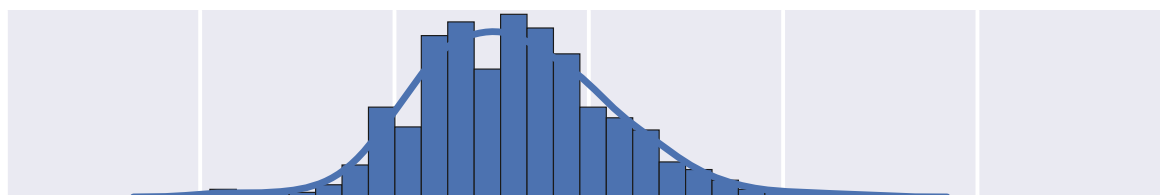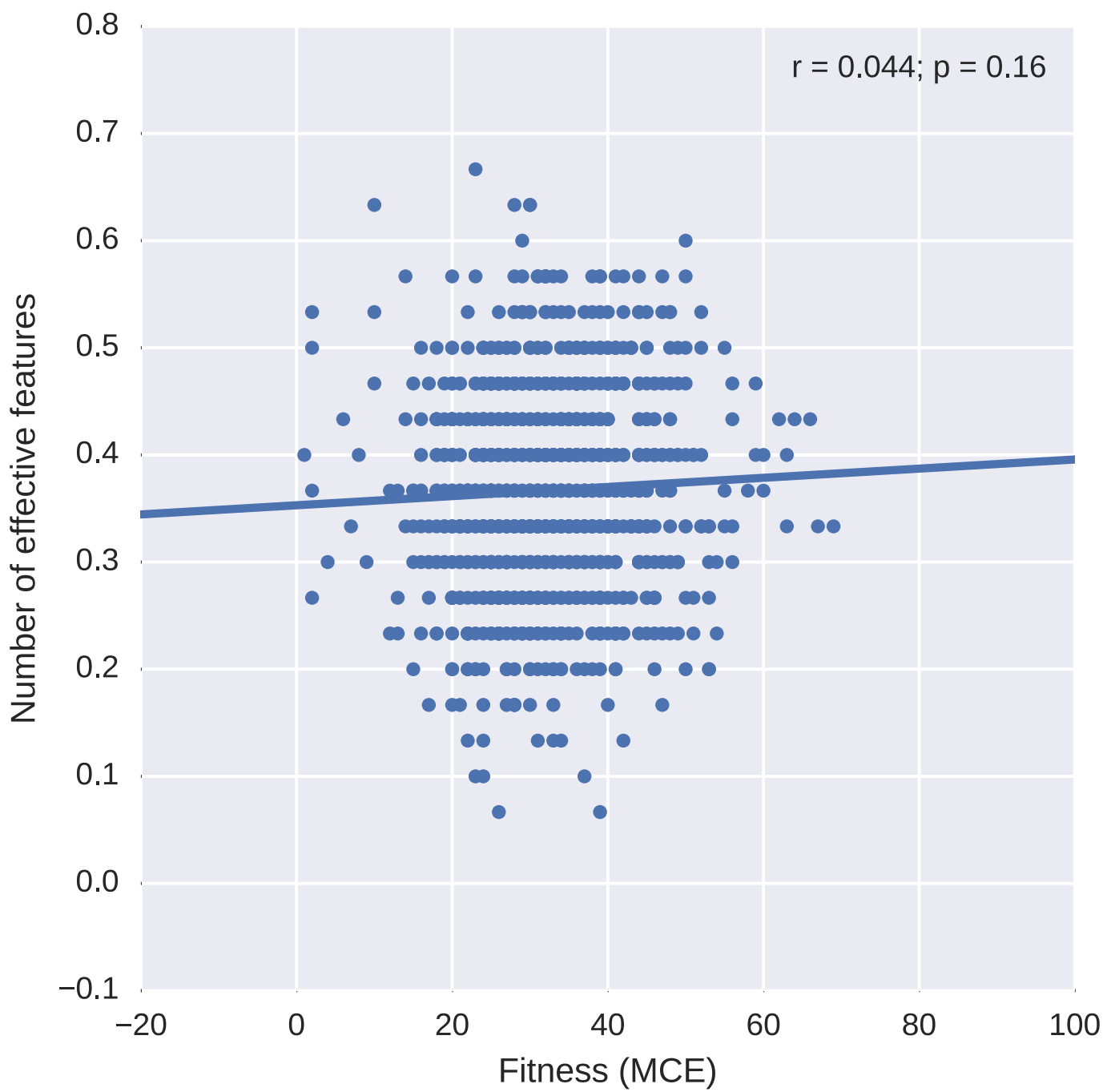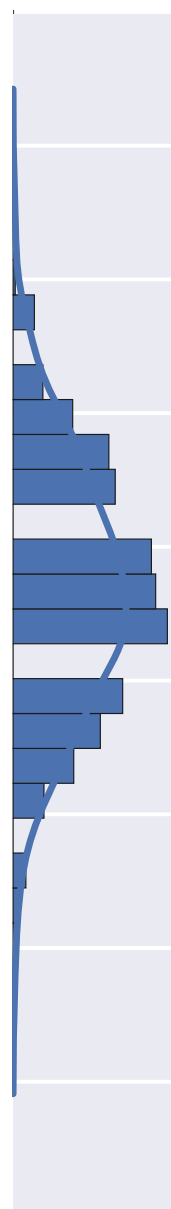

Supplement: S1 Fig — (PDF) [file pcbi.1005986.s001.pdf]

Arg (discovery)

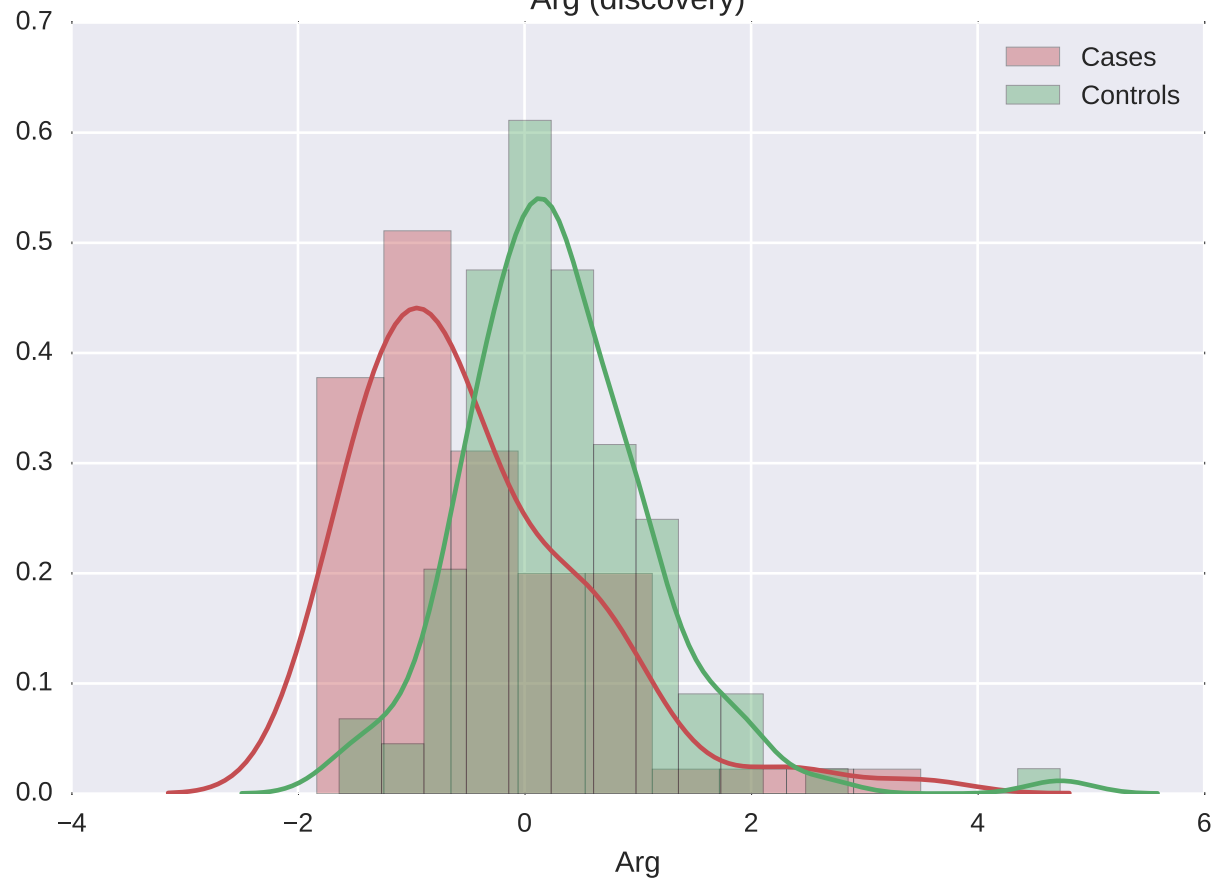

Arg (replication)

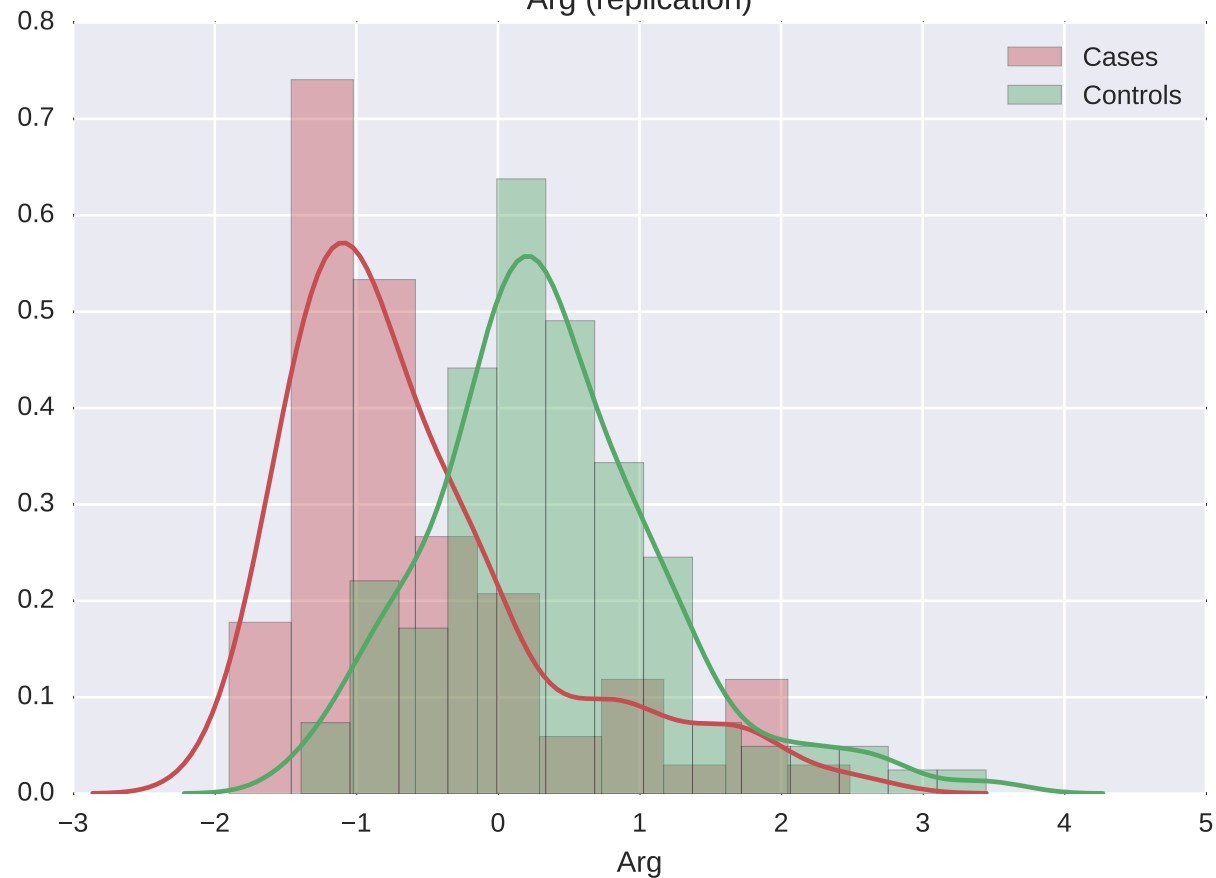

Supplement: S2 Fig — (PDF) [file pcbi.1005986.s002.pdf]

C16 (discovery)

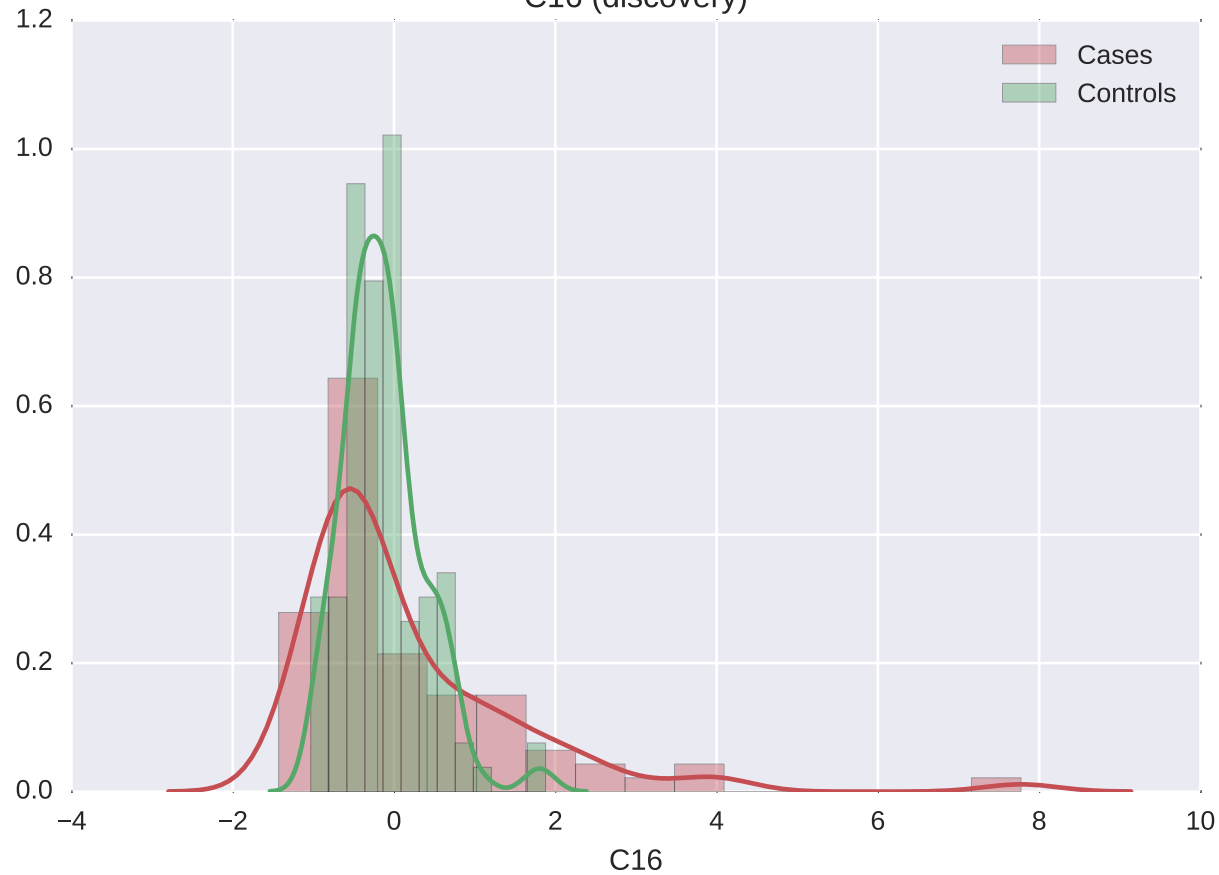

C16 (replication)

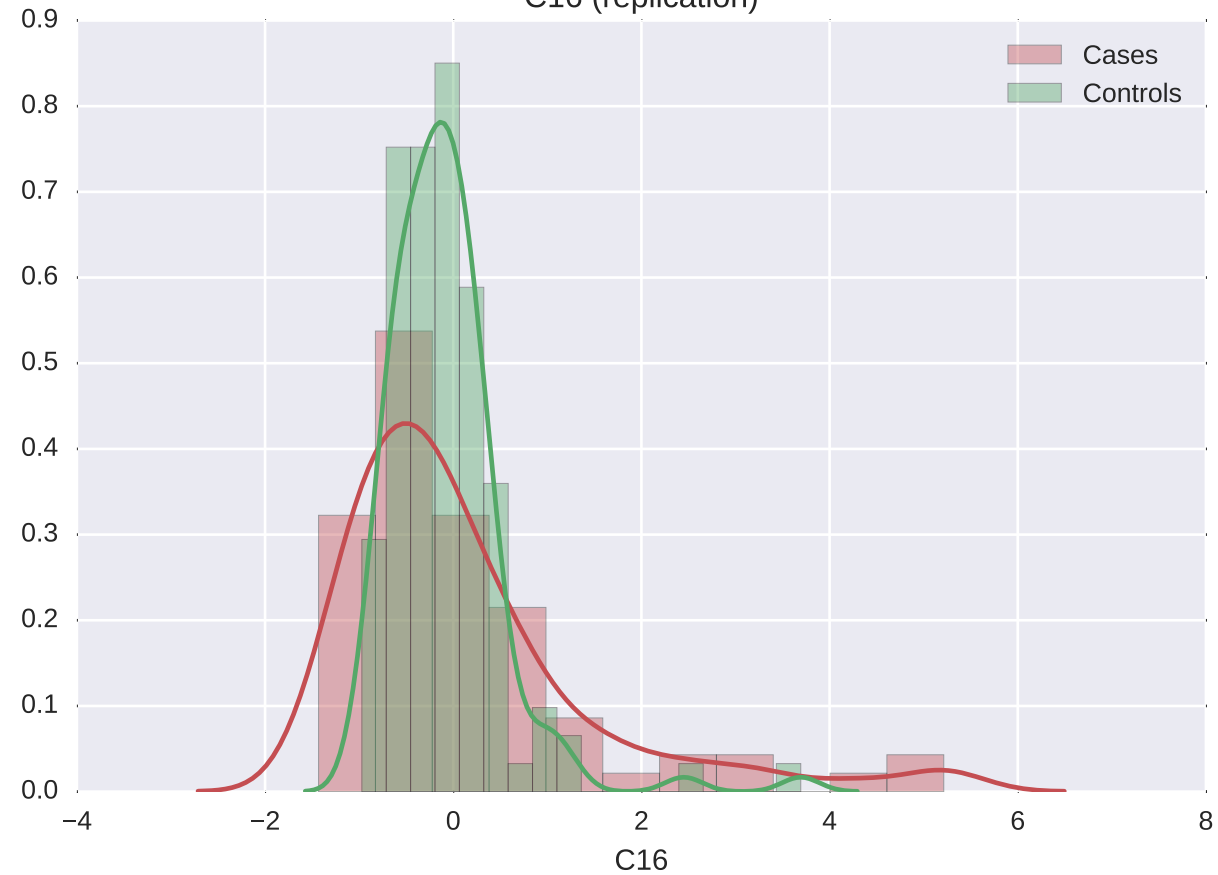

Supplement: S3 Fig — (PDF) [file pcbi.1005986.s003.pdf]

C18:1 (discovery)

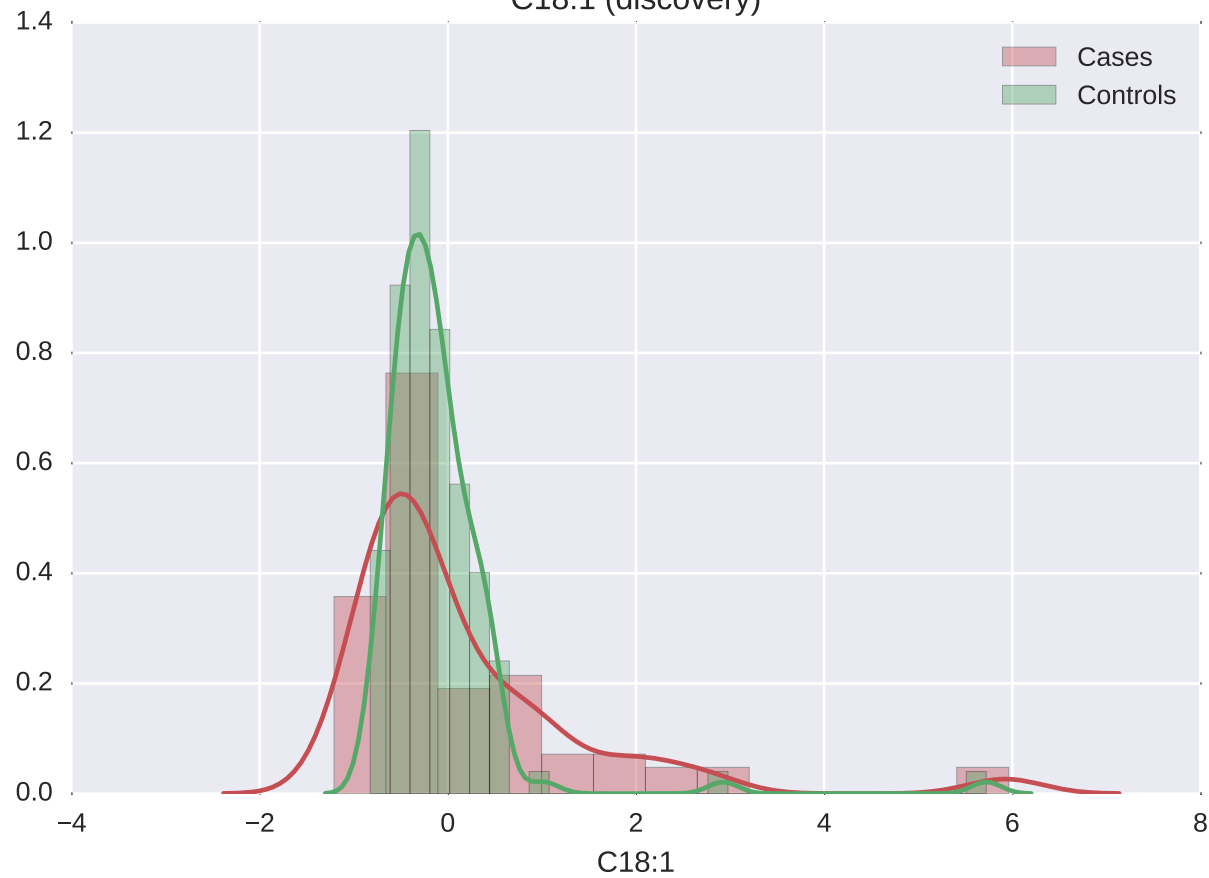

C18:1 (replication)

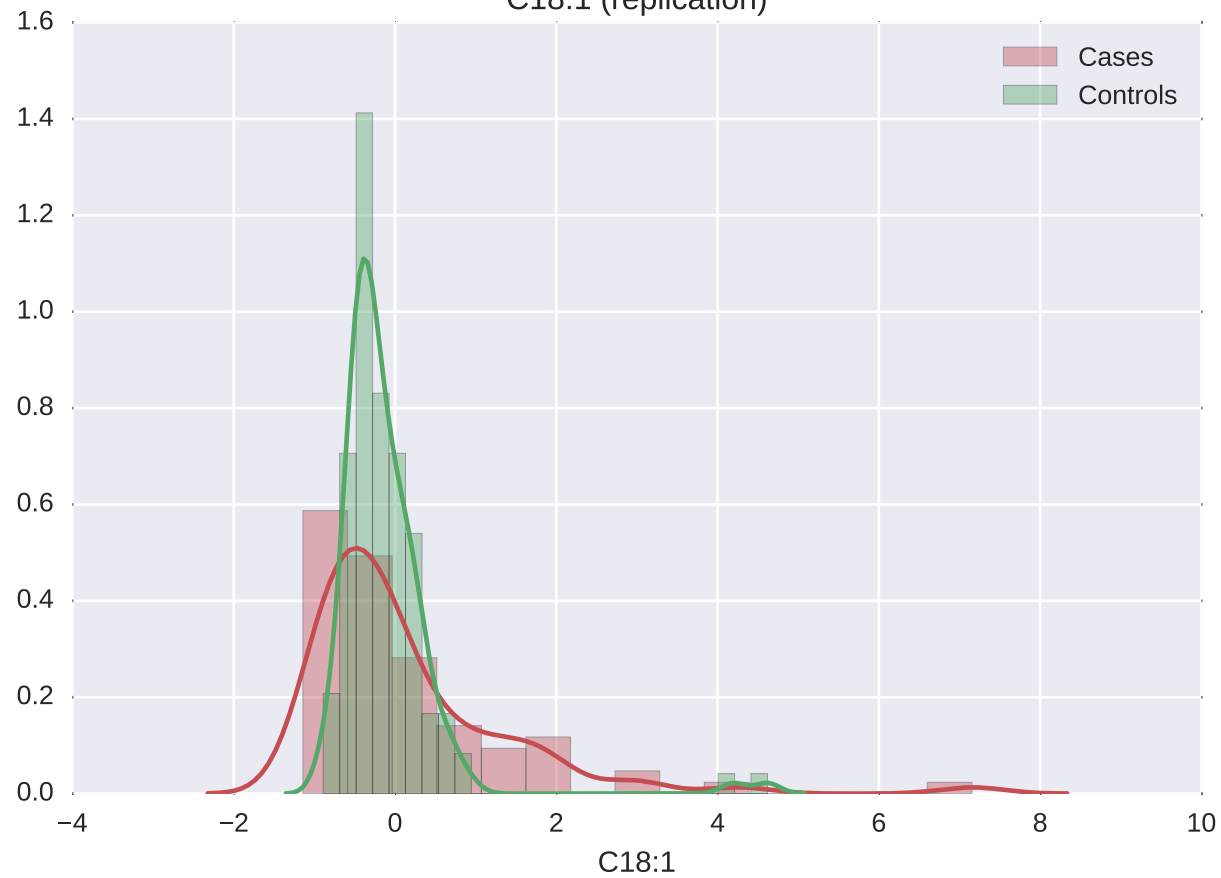

Supplement: S4 Fig — (PDF) [file pcbi.1005986.s004.pdf]

Ile (discovery)

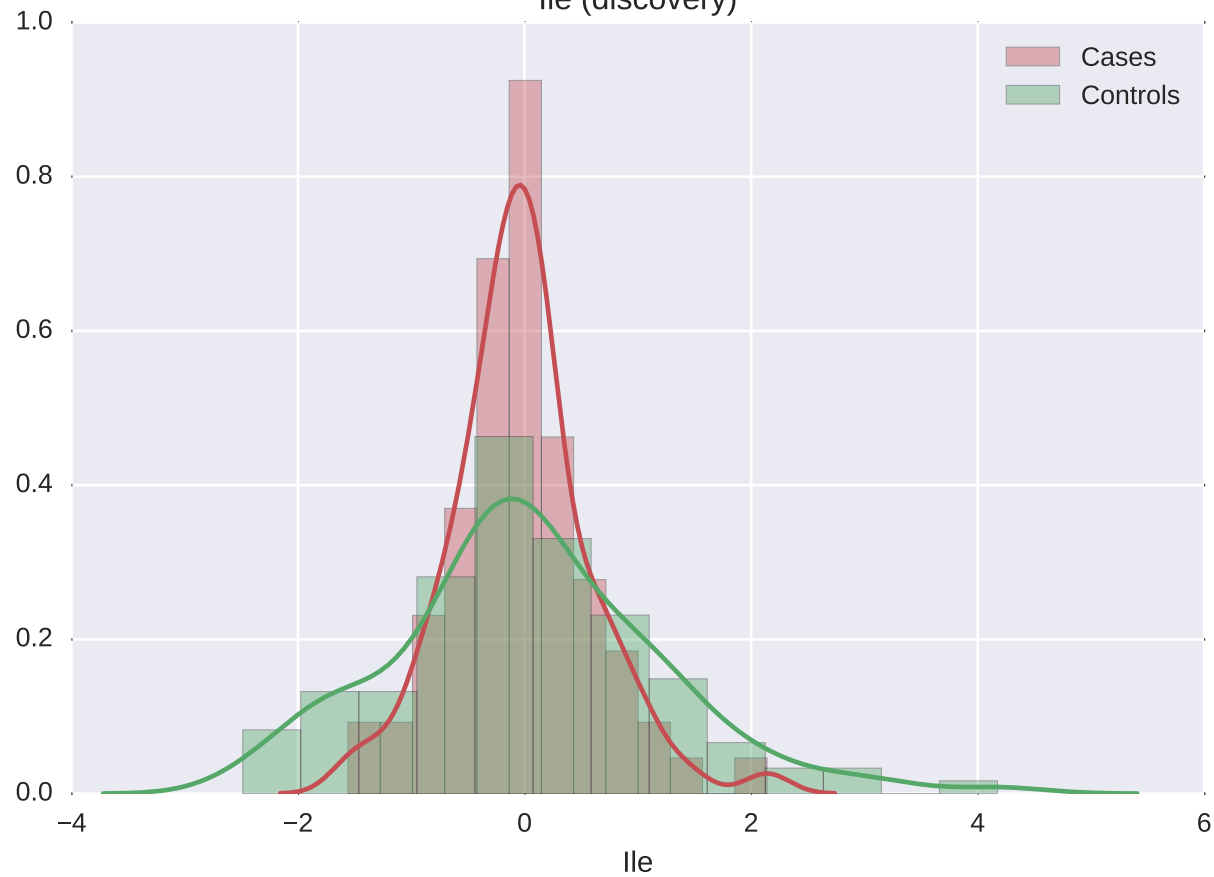

Ile (replication)

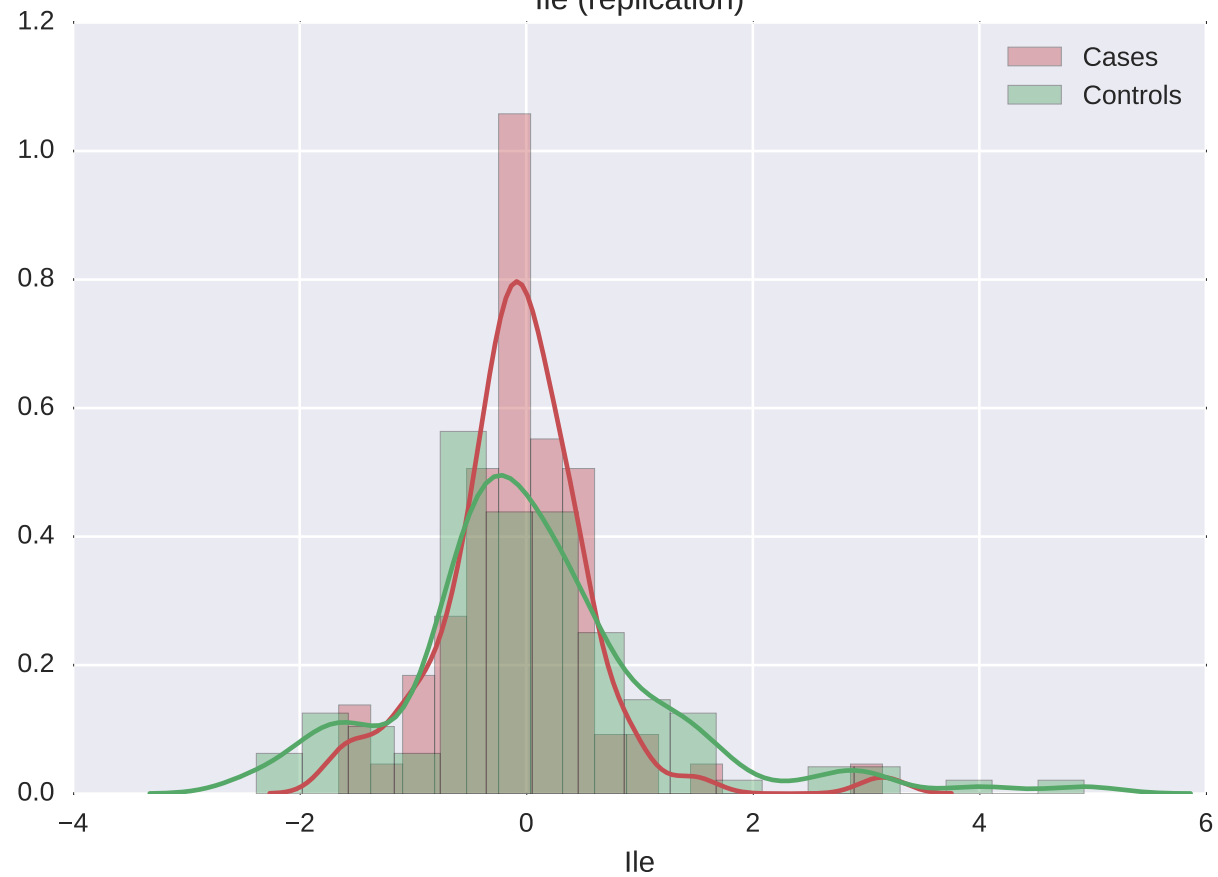

Supplement: S5 Fig — (PDF) [file pcbi.1005986.s005.pdf]

Nitro-Tyr (discovery)

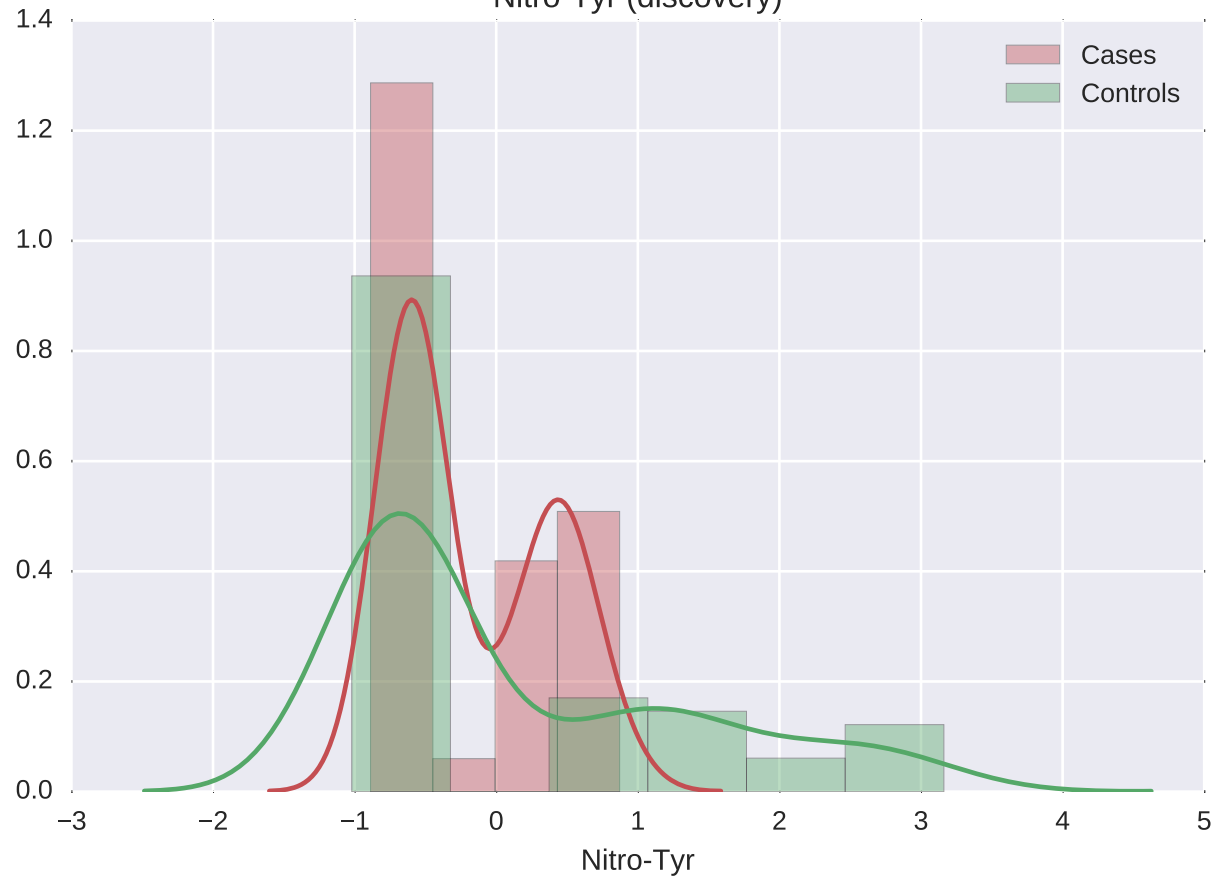

Nitro-Tyr (replication)

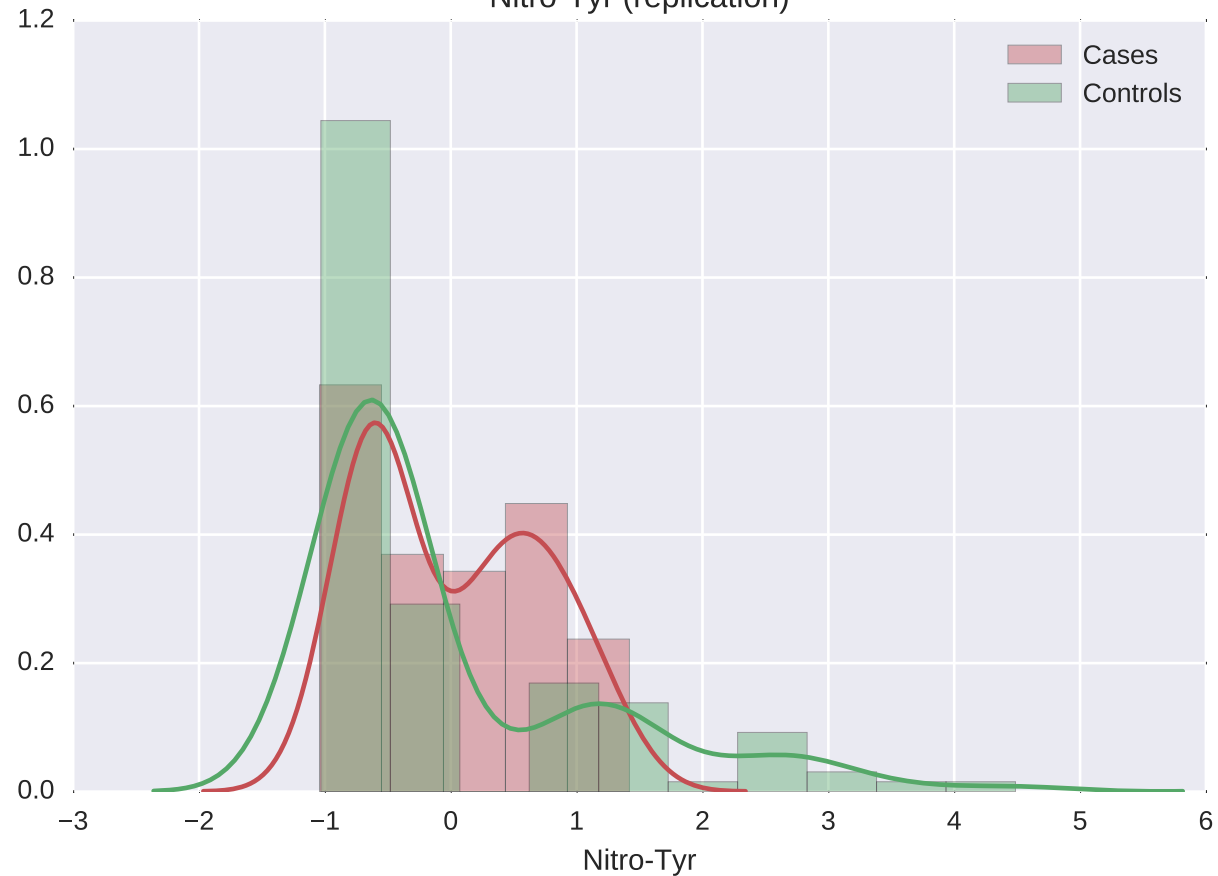

Supplement: S6 Fig — (PDF) [file pcbi.1005986.s006.pdf]

Orn (discovery)

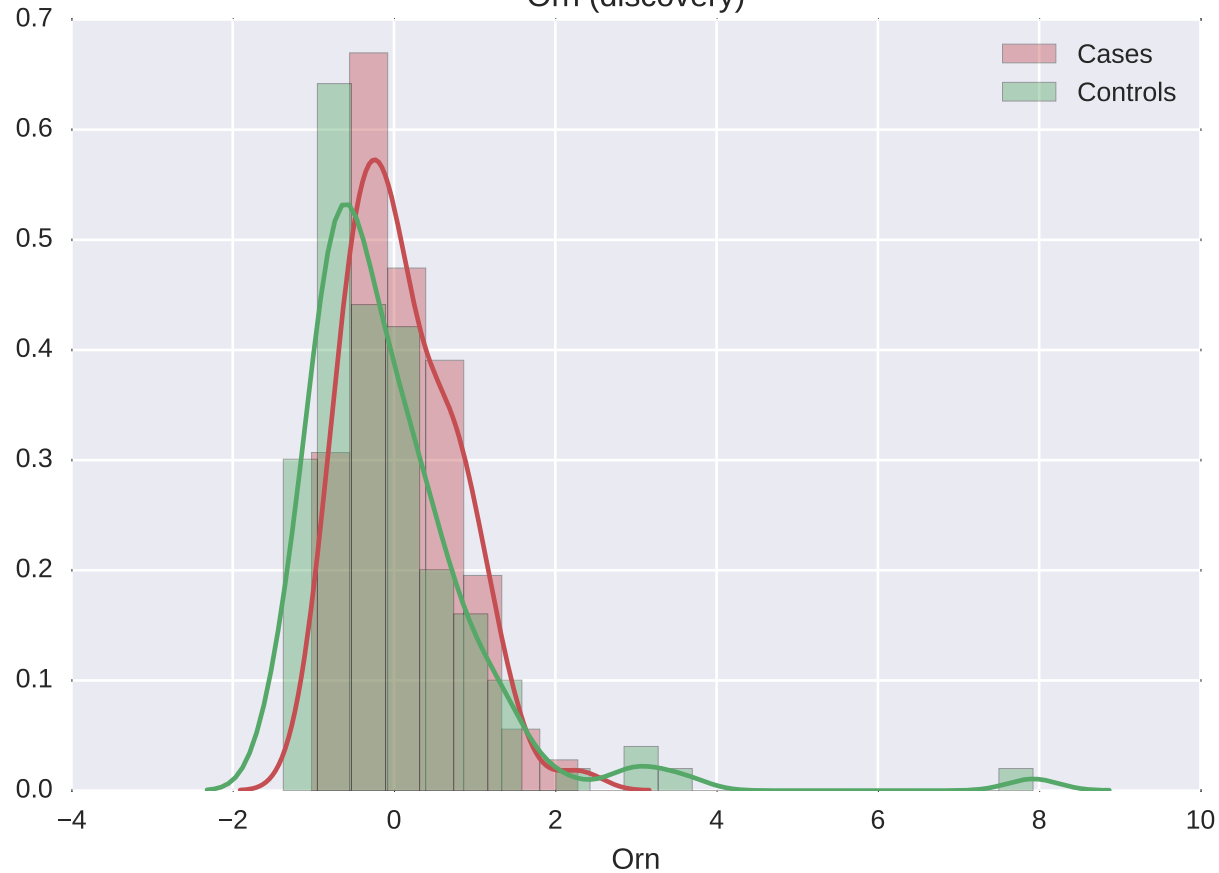

Orn (replication)

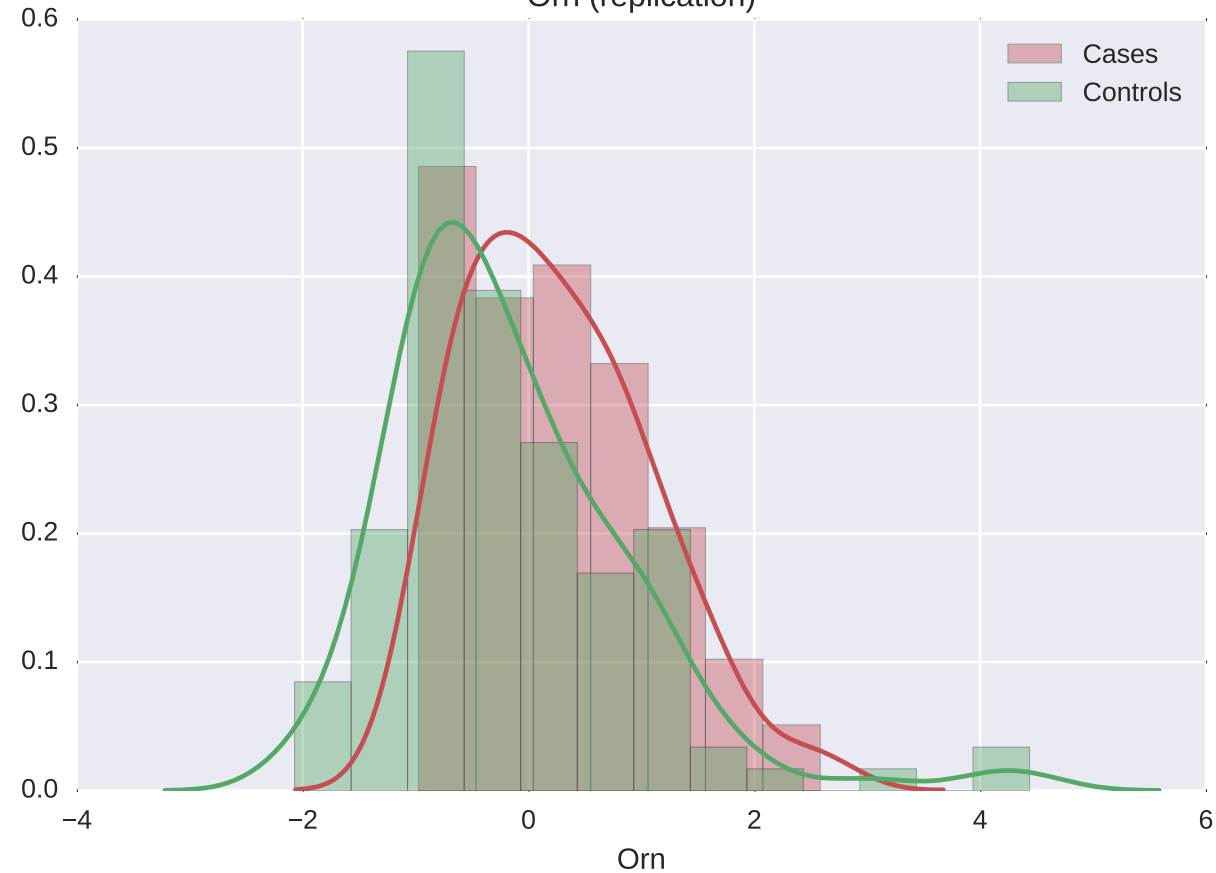

Supplement: S7 Fig — (PDF) [file pcbi.1005986.s007.pdf]

Taurine (discovery)

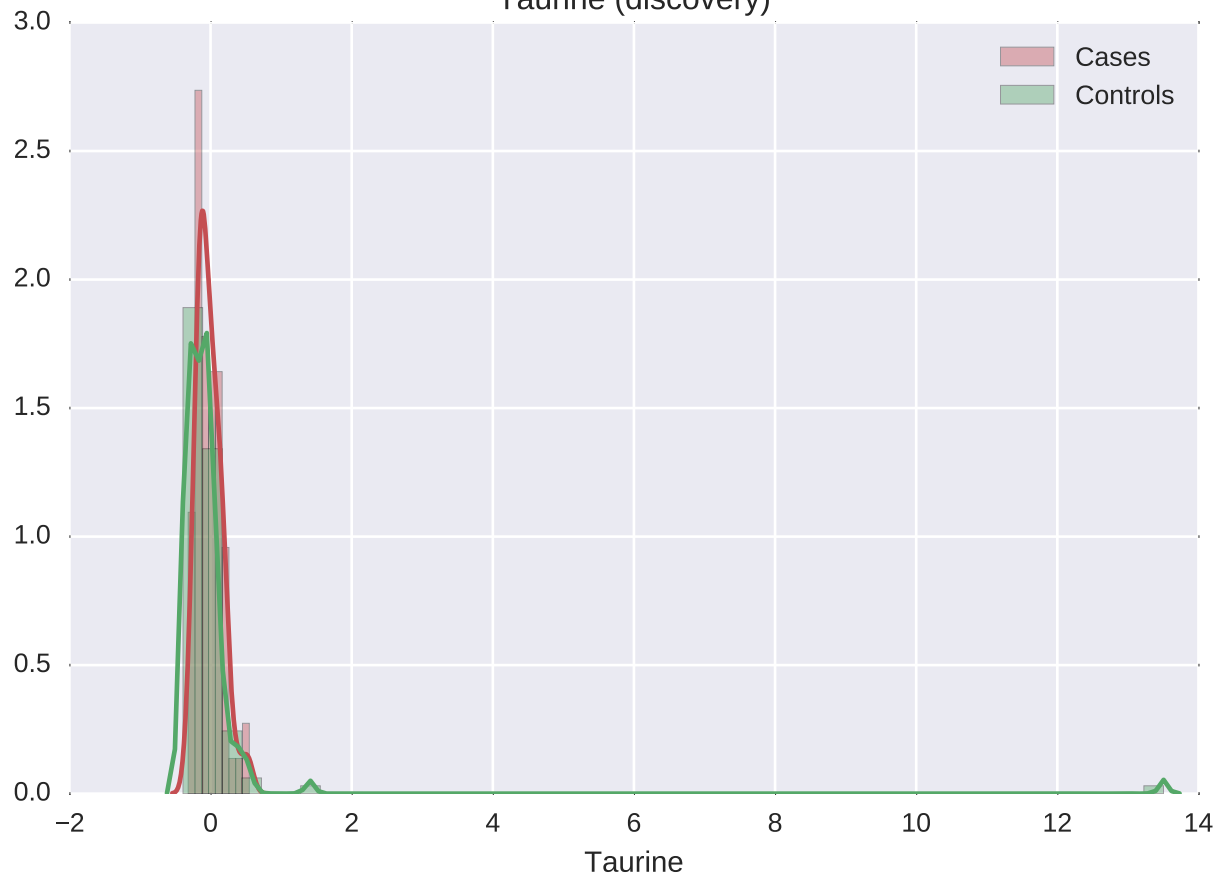

Taurine (replication)

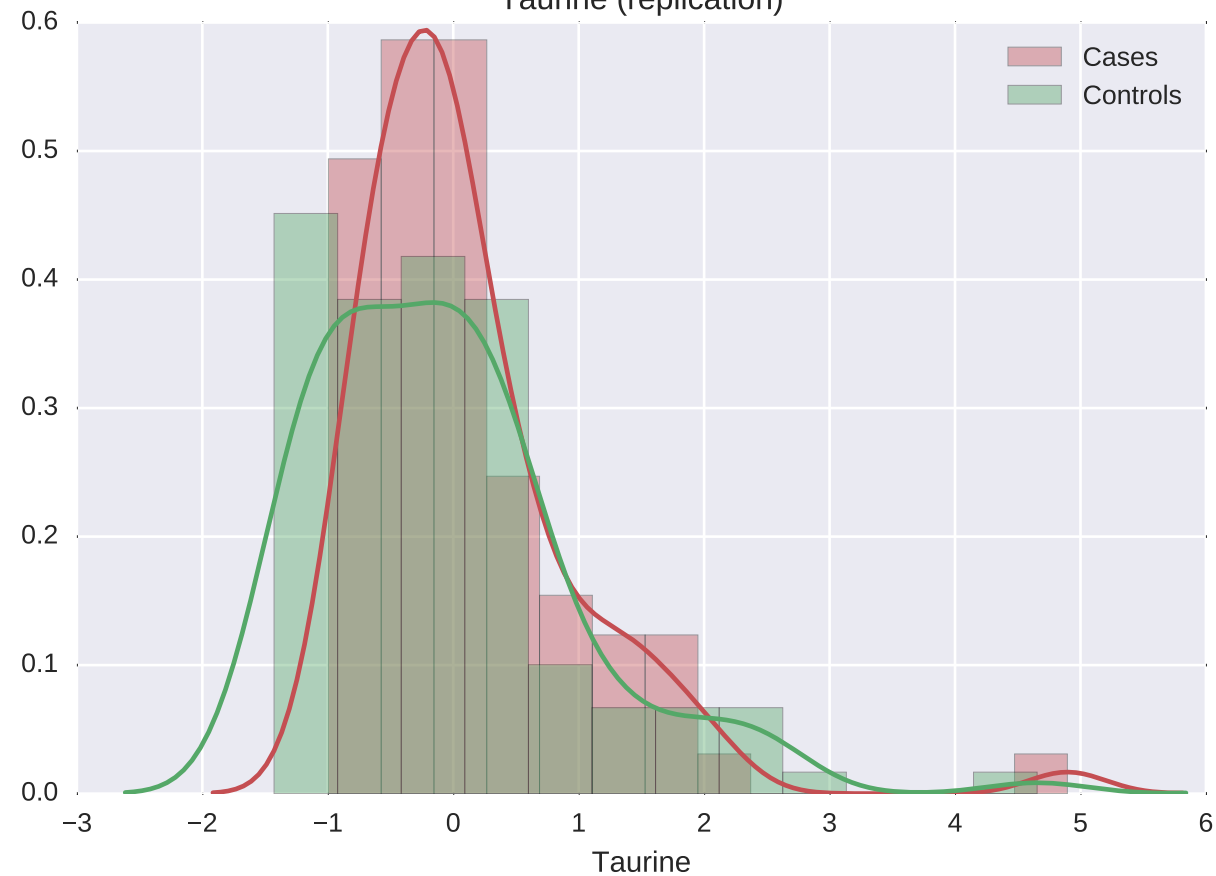

Supplement: S8 Fig — (PDF) [file pcbi.1005986.s008.pdf]

Thr (discovery)

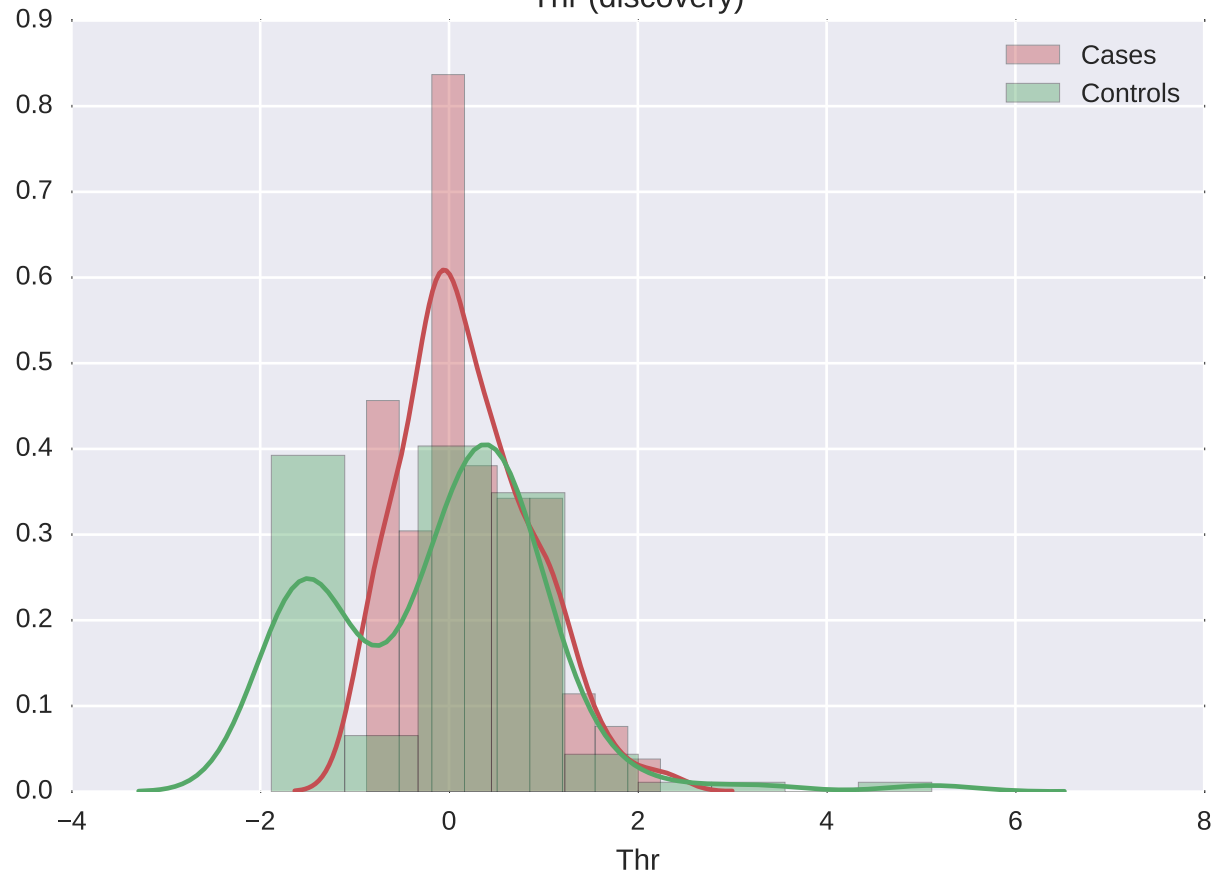

Thr (replication)

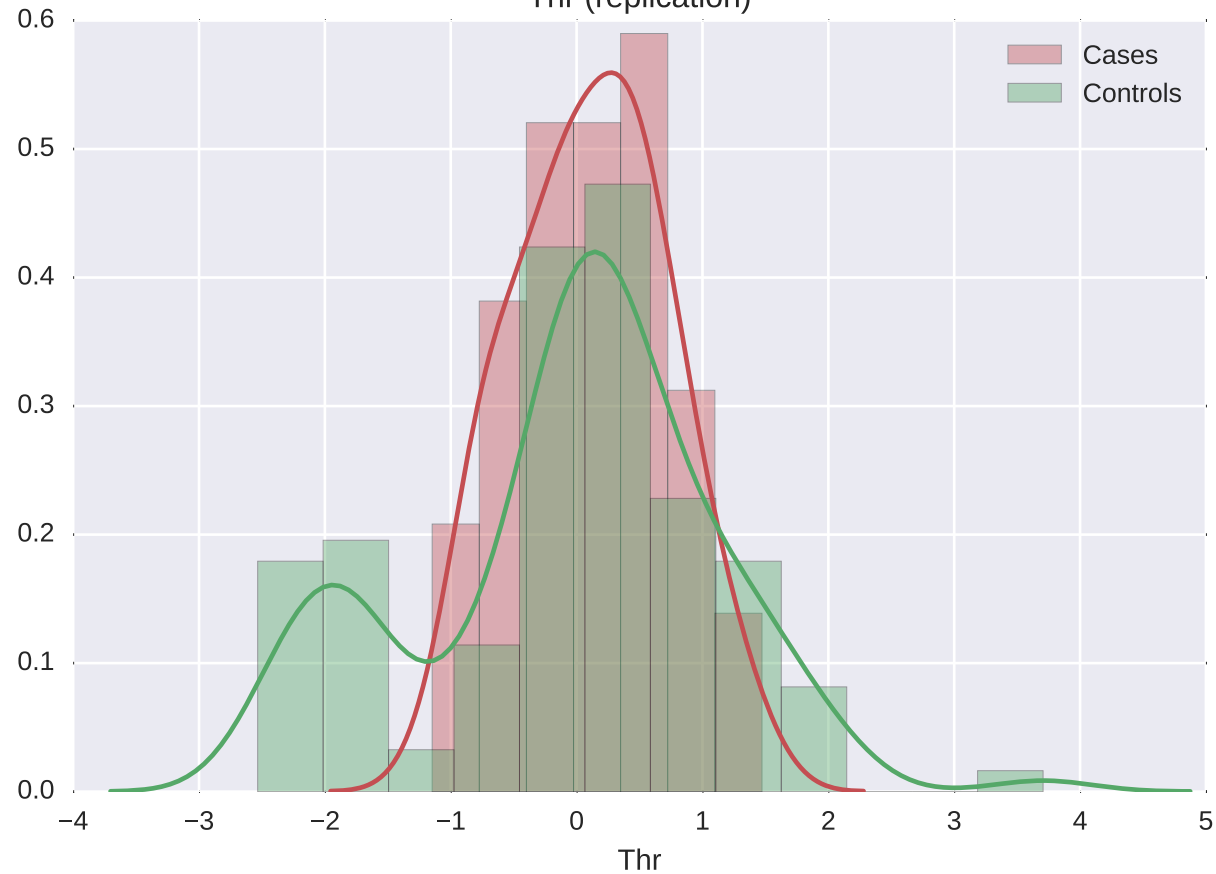

Supplement: S9 Fig — (PDF) [file pcbi.1005986.s009.pdf]

Tyr (discovery)

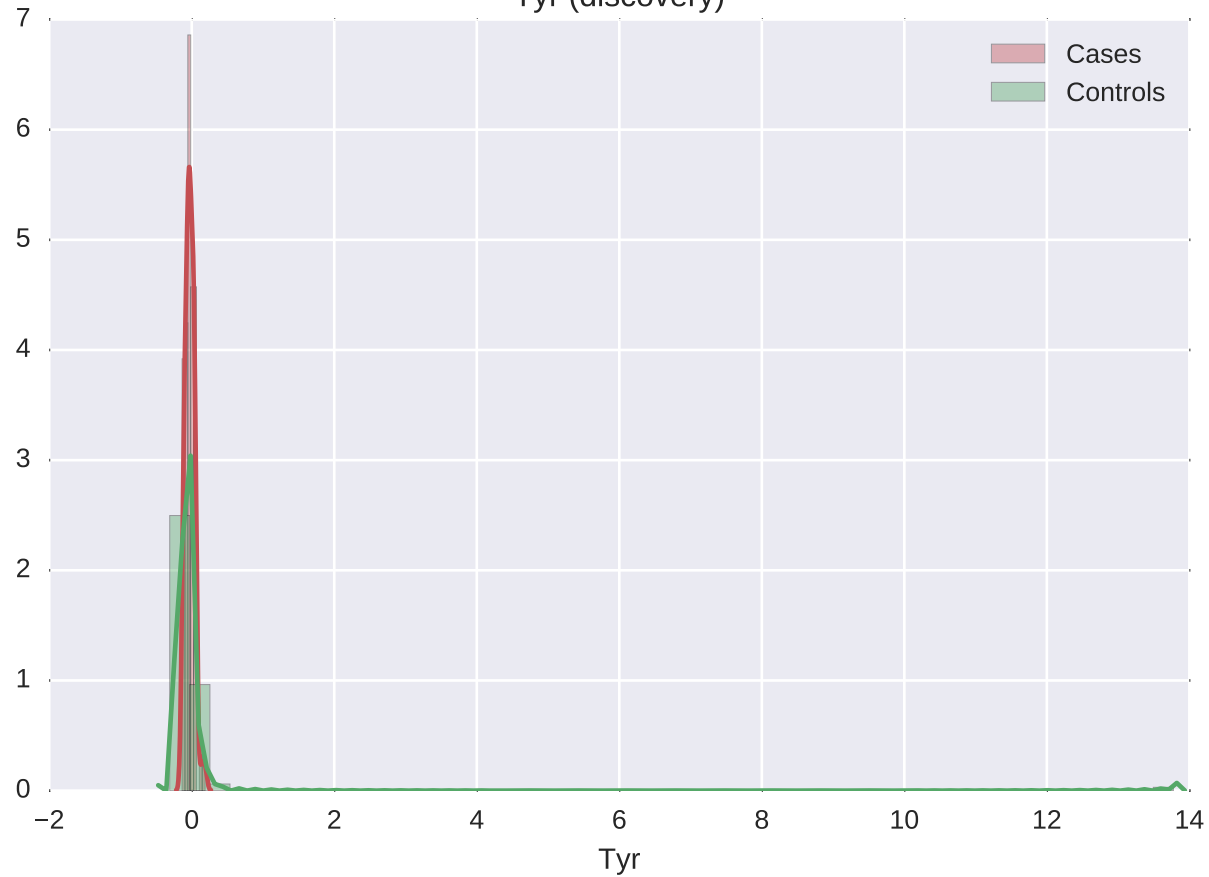

Tyr (replication)

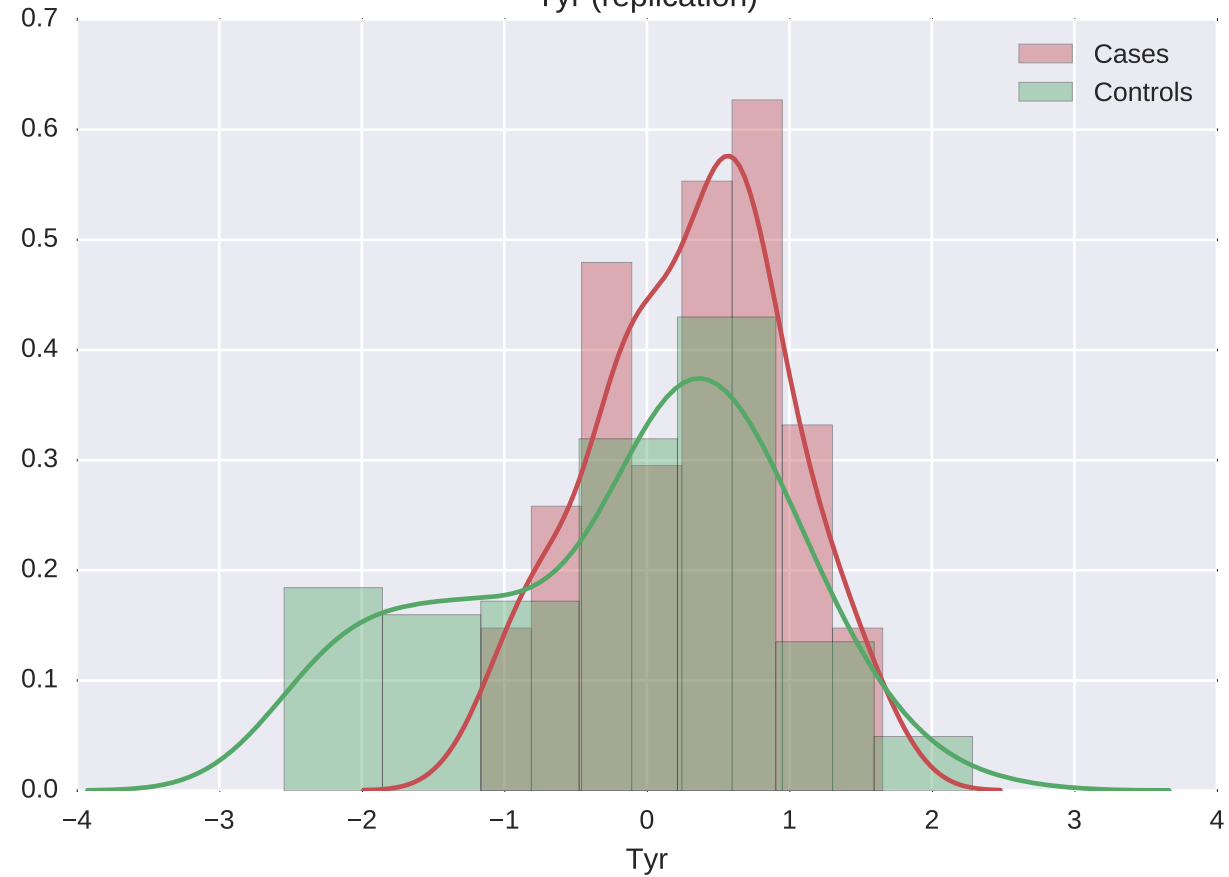

Supplement: S10 Fig — (PDF) [file pcbi.1005986.s010.pdf]
